# Crystal structure of sodium thio­sulfate dihydrate and comparison to the penta­hydrate

**DOI:** 10.1107/S2056989022011975

**Published:** 2023-01-01

**Authors:** Wilhelm Klein

**Affiliations:** a Technische Universität München, School of Natural Sciences, Lichtenbergstrasse 4, 85747 Garching, Germany; University of Aberdeen, United Kingdom

**Keywords:** crystal structure, thio­sulfate, sodium, hydrate, hydrogen bonding

## Abstract

Na_2_S_2_O_3_·2H_2_O has been mentioned in the literature for more than a hundred years and pure samples were prepared and investigated, however, no structural data except for a set of lattice parameters were known to date. Now crystals of this compound have been grown at the surface of an aqueous solution of Na_2_S_2_O_3_ and the structure has been determined at 200 and 100 K. Na_2_S_2_O_3_·2H_2_O crystallizes in the space group *P*2_1_/*n* with two formula units in the asymmetric unit and all atoms occupying general positions. The sodium cations are five- to seven-coordinate by thio­sulfate anions and water mol­ecules and the anions act as mono- and bidentate ligands. In the extended structure, the thio­sulfate anions and water mol­ecules are connected by O—H⋯O and O—H⋯S hydrogen bonds of medium strength to form corrugated layers, which are linked by sodium cations. For comparison, the crystal structure of Na_2_S_2_O_3_·5H_2_O has been determined at the same conditions, *i.e.* for the first time below room temperature.

## Abstract 2

Na_2_S_2_O_3_·2H_2_O has been known for more than a hundred years but no structural data were known to date. Now, crystals of this compound have been grown at the surface of an aqueous solution of Na_2_S_2_O_3_. The sodium cations are five- to seven-coordinate by thio­sulfate anions and water mol­ecules with the anions acting as mono- and bidentate ligands. The thio­sulfate anions and water mol­ecules are connected by O—H⋯O and O—H⋯S hydrogen bonds of medium strength to form corrugated layers, which are linked by sodium cations.

## 1. Chemical context

Thio­sulfates containing the S_2_O_3_^2–^ anion have been studied for more than 150 years (Bunte, 1874 ▸). Nowadays, Na_2_S_2_O_3_ and (NH_4_)_2_S_2_O_3_ are produced on an industrial scale (Barberá *et al.*, 2012 ▸), and the applications of thio­sulfates are growing (Kumar Paul *et al.*, 2009 ▸). One of the most characteristic features of the thio­sulfate anion is the enhanced reactivity including changes of the sulfur oxidation state, which hampered the preparation of pure compounds. For example, the synthesis of pure thio­sulfuric acid succeeded just lately *via* the reaction of Na_2_S_2_O_3_ and anhydrous HF (Hopfinger *et al.*, 2018 ▸), and the first pure thio­sulfate complexes of lanthanides were characterized very recently (Dalton *et al.*, 2021 ▸).

The reactivity also might hinder the preparation of pure anhydrous compounds suitable for structural investigation, and thus, only a few anhydrous thio­sulfate structures are known so far: Na_2_S_2_O_3_ (Sándor & Csordás, 1961 ▸; Teng *et al.*, 1984 ▸), K_2_S_2_O_3_ (Lehner *et al.*, 2013 ▸) and PbS_2_O_3_ (Christensen *et al.*, 1991 ▸). In contrast, numerous hydrates of thio­sulfate compounds have been structurally characterized, and in some classes such as the alkaline-earth metal thio­sulfates, some water mol­ecules of crystallization seem to be crucial for the formation of crystalline matter, indicated by the so far exclusive appearance of hydrated structures *AE*S_2_O_3_·*n*H_2_O with *AE* = Mg (*n* = 6: Elerman *et al.*, 1983 ▸), Ca (*n* = 6: Held & Bohatý, 2004 ▸), Sr (*n* = 5: Held & Bohatý, 2004 ▸; *n* = 1: Klein, 2020 ▸), and Ba (*n* = 1: Manojlović-Muir, 1975 ▸).

The nature of the hydrates in the Na_2_S_2_O_3_ system was intensively studied by Young & Burke (1906 ▸) and by Picon (1924 ▸), who identified either twelve or even fourteen different crystalline hydrates of Na_2_S_2_O_3_, respectively, among them two different dihydrates, by means of their crystalline appearance and by thio­sulfate analysis. The penta­hydrate is by far the most stable compound at ambient conditions, and all other hydrates were found to convert into this phase more or less rapidly. Extended studies of its full dehydration including thermal analyses, Raman spectroscopy and optical microscopy revealed the dihydrate as an inter­mediate phase (Nirsha *et al.*, 1982 ▸; Edwards & Woolf, 1985 ▸; Guarini & Piccini, 1988 ▸). Finally, Edwards and Woolf (1985 ▸) synthesized dihydrate samples with an analytical water content of 1.999 eq. via shaking the penta­hydrate in MeOH at room temperature and presented lattice parameters for a monoclinic cell (*a* = 11.431, *b* = 4.452, *c* = 20.368 Å, *b* = 93.79°, *V* = 1034.4 Å^3^), but no further structural information was given. A different, but unindexed XRD powder pattern was reported for a sample without given composition, which was prepared through dehydration of the penta­hydrate between 338 and 378 K (Nirsha *et al.*, 1982 ▸). Besides these results, the large amount of defined hydrates of Na_2_S_2_O_3_, as implied by the early works, is supported by the structure determinations on single crystals of Na_2_S_2_O_3_·2/3H_2_O (Hesse *et al.*, 1993 ▸), Na_2_S_2_O_3_·5/4H_2_O (Chan *et al.*, 2008 ▸) and Na_2_S_2_O_3_·5H_2_O (Taylor & Beevers, 1952 ▸; Padmanabhan *et al.*, 1971 ▸; Uraz & Armaǧan, 1977 ▸; Lisensky & Levy, 1978 ▸; Prasad & Rani, 2001 ▸). Nevertheless, despite the evidence for its existence, for the dihydrate no structure information is available to date.

For the present paper, the crystal structure of the dihydrate was characterized at 100 and 200 K. For comparison, the structure of the penta­hydrate was determined at the same conditions, *i.e.*, for the first time below ambient temperature.

## 2. Structural commentary

**Figure 1:**
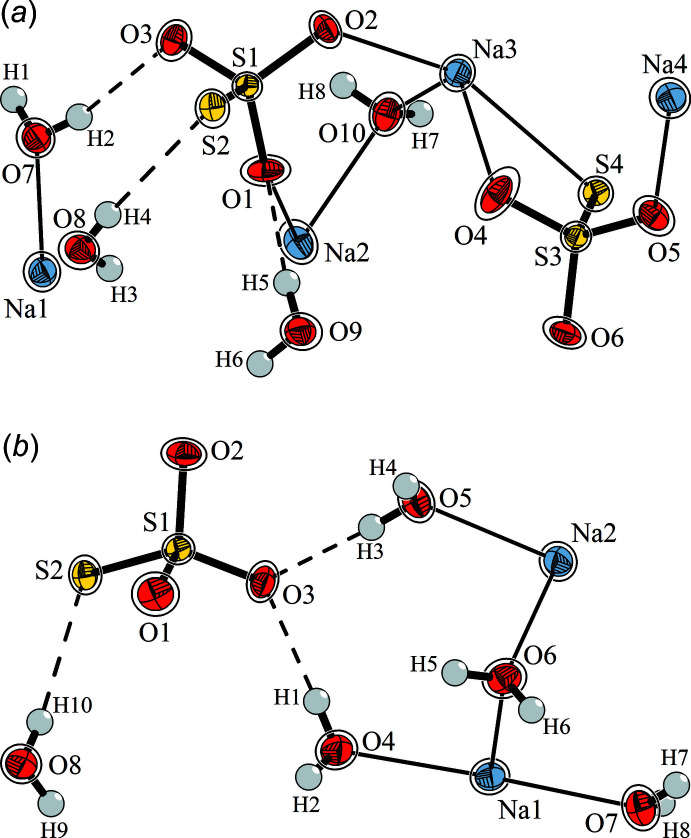
The asymmetric units (*a*) of Na_2_S_2_O_3_·2 H_2_O, and (*b*) of Na_2_S_2_O_3_·5 H_2_O, with a comparison of relative positions and displacement ellipsoids of the non-hydrogen atoms obtained from structure determinations at 100 K (filled atoms) and at 200 K (contours of ellipsoids drawn around filled atoms). Ellipsoids are drawn at the 80% probability level, hydrogen bonds as dashed lines.

The crystal structure of the dihydrate of Na_2_S_2_O_3_ has been determined for the first time. Although this phase has been mentioned in the respective literature for many decades and some sophisticated experiments to synthesize pure samples, usually *via* controlled dehydration of the penta­hydrate, are described, no structural information besides a set of monoclinic lattice parameters is known to date. In the present case, the dihydrate was formed by crystallization at room temperature at the surface of a concentrated aqueous solution, and all dihydrate crystals that have been identified by indexing were isolated from this region. After disturbing the surface tension, most of these crystals subsided immediately to the bottom of the vessel, adding to the bulky crystalline precipitate, which has been identified from X-ray powder patterns as the penta­hydrate without visible impurities. After indexing at room temperature, the crystals were cooled down and datasets were recorded at 200 K and 100 K. Besides slight thermal contraction of lattice parameters and a decrease of displacement parameters (see Fig. 1 ▸ *a*), no structural change has been observed down to 100 K. The same is true for the crystal structure of the penta­hydrate, Na_2_S_2_O_3_·5H_2_O (Fig. 1 ▸ *b*), which has been published formerly and is not discussed here in detail, but was used for comparison. All values mentioned in the structure description below are taken from the structure determinations at 100 K.

**Figure 2:**
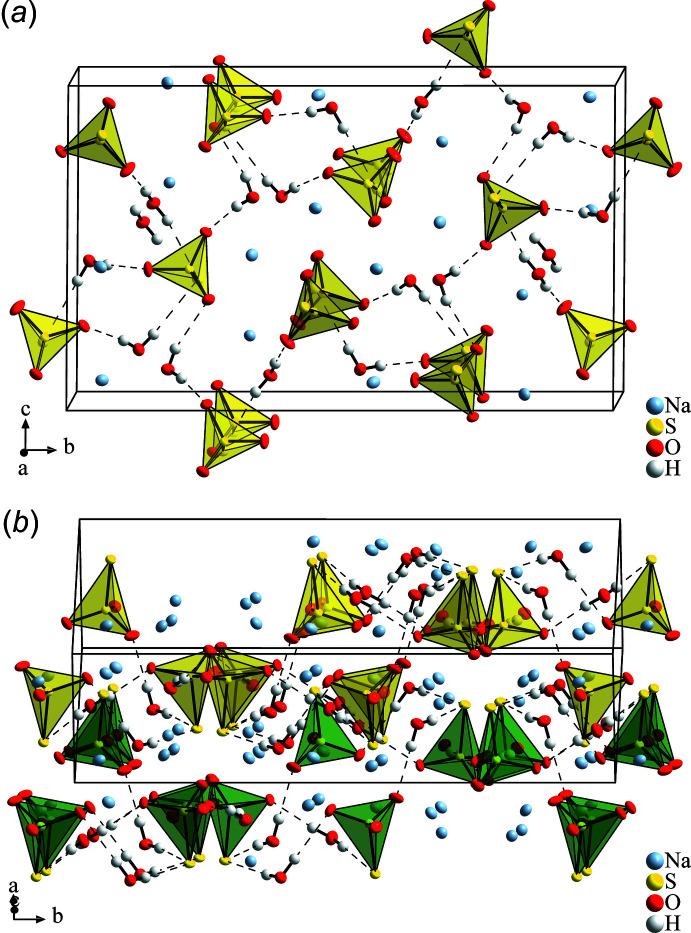
Crystal structure of Na_2_S_2_O_3_·2H_2_O: (*a*) extended unit cell, view along [ 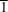 00]; (*b*) section of two layers of S_2_O_3_^2−^ anions and H_2_O mol­ecules connected by hydrogen bonds, view along [ 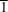 01], differently coloured tetra­hedra belong to different layers. Anisotropic displacement ellipsoids of non-H atoms are drawn with 80% probability, S_2_O_3_^2−^ ions as tetra­hedra, and hydrogen bonds as dashed lines.

Na_2_S_2_O_3_·2H_2_O (Fig. 2 ▸) crystallizes in space group *P*2_1_/*n* with two formula units in the asymmetric unit and all atoms (4 Na, 4 S, 10 O, and 8 H) lying on general positions. The two independent thio­sulfate anions adopt slightly distorted tetra­hedral shapes with average O—S—O angles (110.30°) above and S—S—O angles (108.63°) below the mean bond angle of 109.46°. The S—S bond lengths of 2.0047 (2) Å and 2.0078 (2) Å are similar to that found in the penta­hydrate [2.0266 (1) Å], and, thus, are shorter than the single bond of 2.055 Å in crystalline S_8_ (Rettig & Trotter, 1987 ▸), but substanti­ally longer than the double bond of 1.883 Å in S_2_O (Tiemann *et al.*, 1974 ▸) or 1.889 Å in S_2_ (Pyykkö & Atsumi, 2009 ▸). Also, the S—O bond lengths, which lie between 1.4722 (4) and 1.4841 (4) Å are in the same range as those of the penta­hydrate [1.4665 (4)–1.4867 (4) Å]. The bond-valence sums (Brown & Altermatt, 1985 ▸) for the central sulfur atoms, as calculated with the parameters of Brese & O’Keeffe (1991 ▸), are 5.87 and 5.88 valence units (v.u.) for S1 and S3, respectively, and are in good agreement with a formal charge of +VI as well as with the value of 5.86 v.u. obtained for the corres­ponding S atom in the penta­hydrate. The anions coordinate to the Na^+^ cations and form hydrogen bonds with the water mol­ecules of crystallization: in detail the terminal S and O atoms are surrounded by one Na^+^ and one H_2_O (O1, O4, O6), two Na^+^ and one H_2_O (O2, O3), three Na^+^ (O5), three Na^+^ and two H_2_O (S2), or four Na^+^ and one H_2_O (S4).

**Figure 3:**
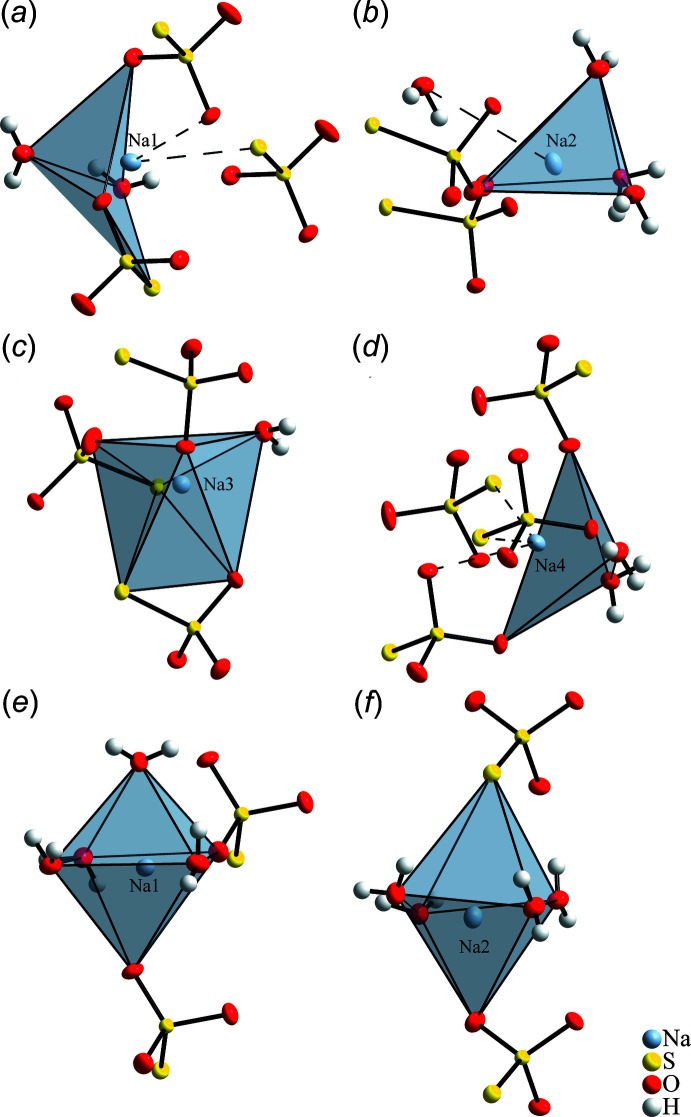
Coordination polyhedra around the Na^+^ cations in Na_2_S_2_O_3_·2H_2_O (*a*–*d*) and in Na_2_S_2_O_3_·5H_2_O (*e*–*f*). Anisotropic displacement ellipsoids of non-H atoms are drawn with 80% probability, weakly or non-coordinating distances above 2.55 Å for Na—O and 3.18 Å for Na—S as dashed lines.

The four independent Na^+^ cations are coordinated irregularly by the S_2_O_3_^2−^ dianions in mono- or bidentate manner and by H_2_O, as illustrated in Fig. 3 ▸ *a*–*d*. The shortest Na—O distances are in the range between 2.3169 (5) Å and 2.4884 (4) Å, with Na—S between 2.9296 (3) and 2.9695 Å. If these environments are considered exclusively, the resulting coordination polyhedra can be inter­preted as an octa­hedron for Na3, mainly distorted due to two S_2_O_3_^2–^ ions coordinating as bidentate ligands, a trigonal prism with one missing corner for Na2 or an octa­hedron with one (Na1) or two (Na4) missing corners. This construction starting from six-vertex polyhedra seems to be justified due to the clearly favoured sixfold coordination for Na^+^ in an environment of oxygen atoms (Gagné & Hawthorne, 2016 ▸). However, for the latter cases of open octa­hedra, S_2_O_3_^2–^ ions as additional ligands with longer bond distances of about 2.5 Å for Na—O and 3.2 Å for Na—S are found, resulting in seven-coordinate polyhedra around Na1 and Na4. For Na2, the H_2_O mol­ecule located above the open side of the polyhedron can be excluded from the coord­ination sphere due to the too large Na—O distance of 3.52 Å and the orientation of the H atoms. The bond-valence sums for the Na cations are 1.08, 1.05, 1.15, and 1.06 v.u. with the highest value for the most conventionally coordinated Na3 ion while reduced values indicate weaker bonds in the coordination spheres of Na1 and Na4 or even an apparently incomplete coordination of Na2. This generally ‘overbonded’ situation for the Na cations as well as the trend to higher values for regular coordination polyhedra is similarly found in the penta­hydrate, the respective values are 1.14 and 1.18 v.u. for the two independent cations in relatively regular octa­hedral coordinations, shown in Fig. 3 ▸ *e*,*f*.

**Figure 4:**
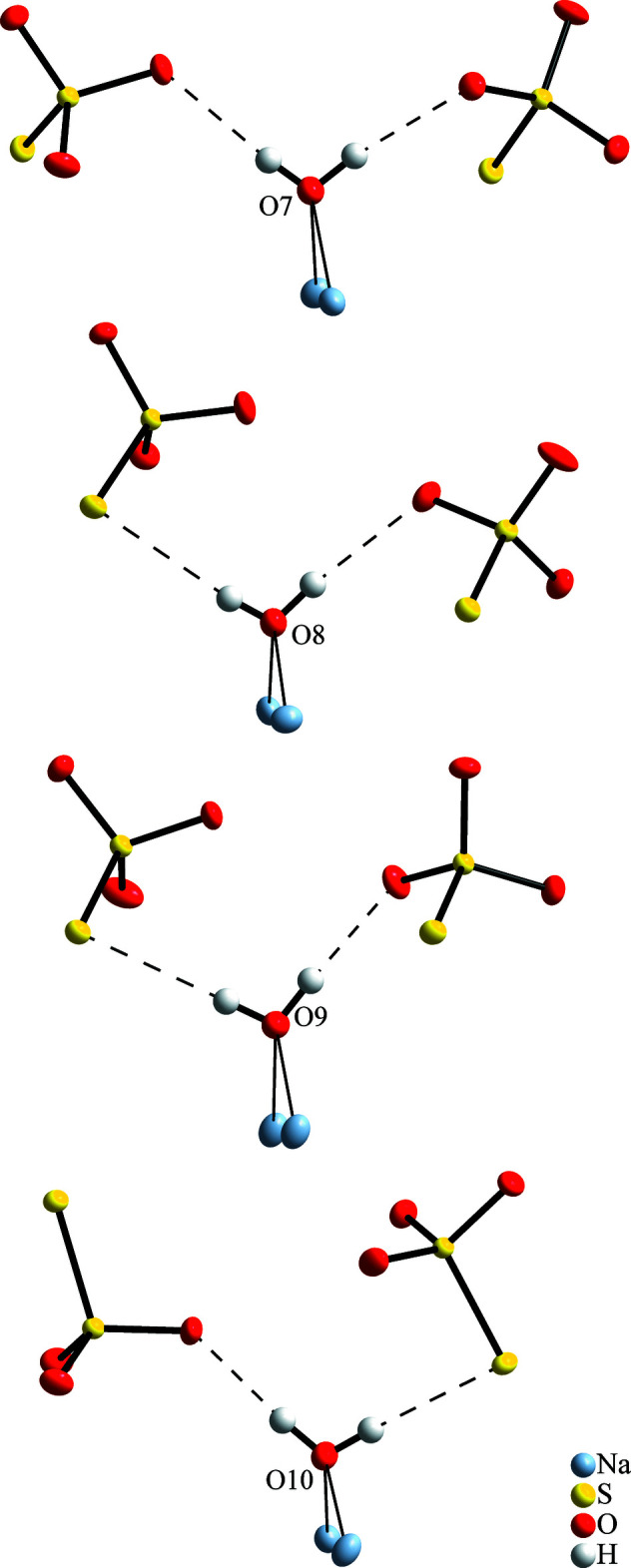
Environments of the crystal water mol­ecules in Na_2_S_2_O_3_·2H_2_O. Anisotropic displacement ellipsoids of non-H atoms are drawn with a probability of 80%, hydrogen bonds as dashed lines, and short contacts to coordinating Na^+^ ions as thin lines.

The four independent water mol­ecules show quite similar, roughly tetra­hedral surroundings, as shown in Fig. 4 ▸. Each H_2_O mol­ecule coordinates to two Na^+^ ions, *i.e.*, as a common vertex of neighbouring coordination polyhedra. All the H atoms form one hydrogen bond of moderate strength with O—H⋯O or O—H⋯S angles above 164°, see Table 1 ▸. This is another similarity to observations in the penta­hydrate, where each H atom is part of one almost linear hydrogen bond (Table 2 ▸).

The highly irregular coordination of the Na^+^ cations in the dihydrate is conspicuous with respect to other more conventional structural features, like the usual bond lengths in the anions or the near-linear hydrogen bonds. Obviously, the structure directing effect of the Na^+^ cations is the weakest among the present building units, although more regular coordination polyhedra, particularly octa­hedra, would have been possible as found in the penta­hydrate as well as in the related structures of Na_6_(S_2_O_3_)_3_·2H_2_O (Hesse *et al.*, 1993 ▸) and Na_8_(S_2_O_3_)_4_·5H_2_O (Chan *et al.*, 2008 ▸). Such open, or at least higher coordinated, polyhedra including weaker bonded ligands as observed in Na_2_S_2_O_3_·2H_2_O should represent an easy possibility to incorporate further water mol­ecules into the structure and, therefore, a hint for the low stability relative to higher hydrates and the retardation of this structure determination.

## 3. Supra­molecular features

**Figure 5:**
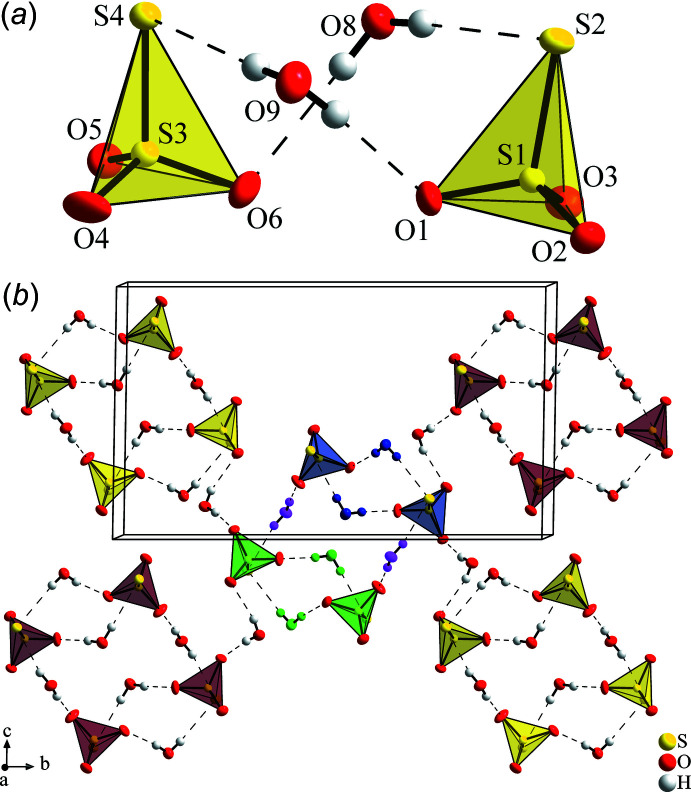
(*a*) A pair of S_2_O_3_^2–^ anions in Na_2_S_2_O_3_·2H_2_O connected by two H_2_O mol­ecules *via* hydrogen bonds. (*b*) Illustration of the hydrogen-bond network between thio­sulfate anions, drawn as tetra­hedra, and water mol­ecules in Na_2_S_2_O_3_·2H_2_O: two S_2_O_3_ tetra­hedra (*e.g.*, the blue ones) are bonded by two H_2_O (blue) to form dimers, which are connected by two H_2_O (pink) with another dimer (green). These tetra­meric units are inter­connected by H_2_O with neighbouring tetra­mers (yellow and red tetra­hedra).

In Na_2_S_2_O_3_·2 H_2_O the thio­sulfate anions and water mol­ecules are connected *via* hydrogen bonds of medium strength, see Table 1 ▸, with all H atoms forming one almost linear bond. Two S_2_O_3_^2–^ ions are connected by two H_2_O mol­ecules to form the building units shown in Fig. 5 ▸ *a*. These dimeric units (*e.g.* blue S_2_O_3_ tetra­hedra and H_2_O mol­ecules in Fig. 5 ▸ *b*) are connected *via* two further H_2_O mol­ecules (pink in Fig. 5 ▸ *b*) with a second dimer (green in Fig. 5 ▸ *b*). The resulting tetra­mers are again inter­linked with neighbouring tetra­mers (yellow and red tetra­hedra in Fig. 5 ▸ *b*) by water mol­ecules, thereby forming corrugated layers lying parallel to (101), also shown in Fig. 2 ▸ *b*. The number of H atoms nicely matches the number of corners of the S_2_O_3_^2–^ tetra­hedra; however, by realizing this connection pattern, six of the eight possible corners of the tetra­hedra dimers accept one hydrogen bond, but one corner (S2) accepts two while one corner (O5) is exclusively surrounded by Na^+^ cations. The layers are not inter­connected by hydrogen bonds but only by Na^+^ cations. This is another difference to the penta­hydrate where the S_2_O_3_^2–^ ions and H_2_O mol­ecules form a three-dimensional framework including hydrogen bonds between water mol­ecules, obviously due to the higher number of H_2_O mol­ecules and, thus, possible hydrogen bonds.

## 4. Database survey

Na_2_S_2_O_3_ and its hydrates have been structurally investigated several times within the second half of the last century. Besides the anhydrous phase (Sándor & Csordás, 1961 ▸; Teng *et al.*, 1984 ▸), including a thorough examination of its temperature dependent polymorphism (von Benda & von Benda, 1979 ▸), some structure determinations of hydrates are reported, namely Na_2_S_2_O_3_·2/3H_2_O (Hesse *et al.*, 1993 ▸), Na_2_S_2_O_3_·5/4H_2_O (Chan *et al.*, 2008 ▸) and Na_2_S_2_O_3_·5H_2_O (Taylor & Beevers, 1952 ▸; Padmanabhan *et al.*, 1971 ▸; Uraz & Armaǧan, 1977 ▸; Lisensky & Levy, 1978 ▸; Prasad & Rani, 2001 ▸), with the sheer number of references obviously illustrating the high stability of the latter phase. For other alkali metal thio­sulfates, the structures of anhydrous K_2_S_2_O_3_ (Lehner *et al.*, 2013 ▸) and K_2_S_2_O_3_·1/3H_2_O (Csordás, 1969 ▸; Chan *et al.*, 2008 ▸; Lehner *et al.*, 2013 ▸) as well as of the monohydrates of Rb_2_S_2_O_3_ (Lehner *et al.*, 2013 ▸) and Cs_2_S_2_O_3_ (Winkler *et al.*, 2016 ▸) have been reported.

## 5. Synthesis and crystallization

Colourless crystals of Na_2_S_2_O_3_·2H_2_O were grown at ambient conditions from an aqueous solution of Na_2_S_2_O_3_. The crystals were found floating at the surface of the mother liquor, but sank down to the bottom of the crystallization vessel immediately after disturbing the surface tension. A batch of crystals was immersed into perfluoro­ether, and the crystals were found to be unscathed and stable at room temperature for days. In contrast, no crystals of the dihydrate could be found from the crystal bulk at the bottom of the vessel, but all crystals isolated later from there were penta­hydrate crystals. In addition, an X-ray powder pattern of a sample prepared from this bulk did not contain any other reflections than those of the penta­hydrate.

## 6. Refinement

Crystal data, data collection, and structure refinement details are summarized in Table 3 ▸. In all presented structure refinements, all hydrogen atoms could be located from the difference-Fourier map and were refined with free atomic coordinates and isotropic displacement parameters.

## Supplementary Material

Crystal structure: contains datablock(s) global, Na2S2O3H2O2_100K, Na2S2O3H2O2_200K, Na2S2O3H2O5_100K, Na2S2O3H2O5_200K. DOI: 10.1107/S2056989022011975/hb8046sup1.cif

Structure factors: contains datablock(s) Na2S2O3H2O2_100K. DOI: 10.1107/S2056989022011975/hb8046Na2S2O3H2O2_100Ksup2.hkl

Structure factors: contains datablock(s) Na2S2O3H2O2_200K. DOI: 10.1107/S2056989022011975/hb8046Na2S2O3H2O2_200Ksup3.hkl

Structure factors: contains datablock(s) Na2S2O3H2O5_100K. DOI: 10.1107/S2056989022011975/hb8046Na2S2O3H2O5_100Ksup4.hkl

Structure factors: contains datablock(s) Na2S2O3H2O5_200K. DOI: 10.1107/S2056989022011975/hb8046Na2S2O3H2O5_200Ksup5.hkl

CCDC references: 2227273, 2227272, 2227271, 2227270

Additional supporting information:  crystallographic information; 3D view; checkCIF report

## Figures and Tables

**Table 1:** Hydrogen-bond geometry (Å, °) for Na_2_S_2_O_3_·2H_2_O at 100 K

*D*—H⋯*A*	*D*—H	H⋯*A*	*D*⋯*A*	*D*—H⋯*A*
O7—H1⋯O2^i^	0.844 (11)	2.090 (11)	2.9246 (5)	170.1 (11)
O7—H2⋯O3	0.782 (12)	2.190 (12)	2.9498 (6)	164.3 (12)
O8—H3⋯O6^ii^	0.827 (12)	2.211 (12)	3.0282 (6)	170.1 (11)
O8—H4⋯S2	0.772 (14)	2.545 (14)	3.3142 (4)	174.5 (13)
O9—H5⋯O1	0.851 (15)	1.966 (15)	2.8142 (6)	174.7 (15)
O9—H6⋯S4^ii^	0.825 (13)	2.471 (13)	3.2959 (4)	177.9 (12)
O10—H7⋯O4^iii^	0.828 (13)	1.936 (13)	2.7585 (5)	172.4 (12)
O10—H8⋯S2^iii^	0.810 (13)	2.421 (13)	3.2183 (4)	168.3 (12)

Symmetry codes: (i) 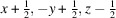 ; (ii) 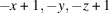 ; (iii) 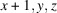 .

**Table 2:** Hydrogen-bond geometry (Å, °) for Na_2_S_2_O_3_·5H_2_O at 100 K

*D*—H⋯*A*	*D*—H	H⋯*A*	*D*⋯*A*	*D*—H⋯*A*
O4—H1⋯O3	0.808 (12)	2.001 (12)	2.8067 (5)	176.1 (13)
O4—H2⋯O2^i^	0.812 (12)	2.017 (12)	2.8175 (5)	168.6 (12)
O5—H3⋯O3	0.820 (13)	1.973 (13)	2.7912 (5)	174.9 (12)
O5—H4⋯O3^ii^	0.809 (13)	2.074 (13)	2.8736 (5)	169.2 (12)
O6—H5⋯O4^ii^	0.847 (14)	1.993 (14)	2.8365 (6)	173.8 (13)
O6—H6⋯S2^iii^	0.850 (13)	2.495 (13)	3.3404 (4)	173.6 (12)
O7—H7⋯S2^iv^	0.824 (13)	2.527 (13)	3.3356 (4)	167.5 (11)
O7—H8⋯O8^v^	0.812 (13)	2.017 (13)	2.8280 (6)	177.2 (12)
O8—H9⋯S2^i^	0.792 (13)	2.554 (13)	3.3147 (4)	161.6 (12)
O8—H10⋯S2	0.785 (14)	2.558 (14)	3.3381 (4)	173.0 (13)

Symmetry codes: (i) 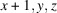 ; (ii) 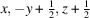 ; (iii) 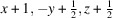 ; (iv) 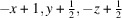 ; (v) 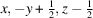 .

**Table 3:** Experimental details

	Na_2_S_2_O_3_·2H_2_O at 100 K	Na_2_S_2_O_3_·2H_2_O at 200 K	Na_2_S_2_O_3_·5H_2_O at 100 K	Na_2_S_2_O_3_·5H_2_O at 200 K
Crystal data				
Chemical formula	Na_2_S_2_O_3_·2H_2_O	Na_2_S_2_O_3_·2H_2_O	Na_2_S_2_O_3_·5H_2_O	Na_2_S_2_O_3_·5H_2_O
*M*_r_	194.13	194.13	248.18	248.18
Crystal system, space group	Monoclinic, *P*2_1_/*n*	Monoclinic, *P*2_1_/*n*	Monoclinic, *P*2_1_/*c*	Monoclinic, *P*2_1_/*c*
Temperature (K)	100	200	100	200
*a*, *b*, *c* (Å)	5.7719 (1), 19.3257 (3), 11.5162 (3)	5.8003 (1), 19.3713 (4), 11.5520 (3)	5.9187 (1), 21.5173 (4), 7.4979 (1)	5.9357 (1), 21.5424 (7), 7.5026 (2)
β (°)	102.388 (2)	102.331 (2)	103.722 (1)	103.722 (2)
*V* (Å^3^)	1254.68 (4)	1268.03 (5)	927.64 (3)	931.97 (4)
*Z*	8	8	4	4
Radiation type	Mo *K*α	Mo *K*α	Mo *K*α	Mo *K*α
μ (mm^−1^)	0.93	0.92	0.67	0.67
Crystal size (mm)	0.25 × 0.2 × 0.15	0.25 × 0.2 × 0.15	0.6 × 0.3 × 0.15	0.6 × 0.3 × 0.15

Data collection				
Diffractometer	Stoe StadiVari	Stoe StadiVari	Stoe StadiVari	Stoe StadiVari
Absorption correction	Empirical (using intensity measurements) (*X-AREA*; Stoe & Cie, 2015 ▸)	Empirical (using intensity measurements) (*X-AREA*; Stoe & Cie, 2015 ▸)	Empirical (using intensity measurements) (*X-AREA*; Stoe & Cie, 2015 ▸)	Empirical (using intensity measurements) (*X-AREA*; Stoe & Cie, 2015 ▸)
*T*_min_, *T*_max_	0.919, 1.000	0.920, 1.000	0.868, 1.000	0.869, 1.000
No. of measured, independent and observed [*I* > 2σ(*I*)] reflections	52592, 8735, 7524	55208, 8848, 7181	64240, 6486, 5525	56700, 5006, 4212
*R*_int_	0.019	0.024	0.026	0.023
(sin θ/λ)_max_ (Å^−1^)	0.946	0.948	0.948	0.869

Refinement				
*R*[*F* ^2^ > 2σ(*F* ^2^)], *wR*(*F* ^2^), *S*	0.017, 0.047, 1.02	0.022, 0.057, 1.01	0.020, 0.047, 1.06	0.019, 0.052, 1.08
No. of reflections	8735	8848	6486	5006
No. of parameters	195	195	149	149
H-atom treatment	All H-atom parameters refined	All H-atom parameters refined	All H-atom parameters refined	All H-atom parameters refined
Δρ_max_, Δρ_min_ (e Å^−3^)	0.55, −0.41	0.58, −0.34	0.54, −0.27	0.42, −0.28

Computer programs: *X-AREA* (Stoe & Cie, 2015 ▸), *SHELXS97* (Sheldrick, 2008 ▸), *SHELXL2014/7* (Sheldrick, 2015 ▸) and *DIAMOND* (Brandenburg & Putz, 2012 ▸).

## supplementary crystallographic information

### Sodium thiosulfate dihydrate (Na2S2O3H2O2_100K) Crystal data

**Table:**

Na_2_S_2_O_3_·2H_2_O	*F*(000) = 784
*M**_r_* = 194.13	*D*_x_ = 2.055 Mg m^−^^3^
Monoclinic, *P*2_1_/*n*	Mo *K*α radiation, λ = 0.71073 Å
*a* = 5.7719 (1) Å	Cell parameters from 74172 reflections
*b* = 19.3257 (3) Å	θ = 3.6–42.6°
*c* = 11.5162 (3) Å	µ = 0.93 mm^−^^1^
β = 102.388 (2)°	*T* = 100 K
*V* = 1254.68 (4) Å^3^	Block, colourless
*Z* = 8	0.25 × 0.2 × 0.15 mm

### Sodium thiosulfate dihydrate (Na2S2O3H2O2_100K) Data collection

**Table:**

Stoe StadiVari diffractometer	8735 independent reflections
Radiation source: Genix 3D HF Mo	7524 reflections with *I* > 2σ(*I*)
Graded multilayer mirror monochromator	*R*_int_ = 0.019
Detector resolution: 5.81 pixels mm^-1^	θ_max_ = 42.3°, θ_min_ = 3.6°
ω scans	*h* = −10→10
Absorption correction: empirical (using intensity measurements) (X-AREA; Stoe & Cie, 2015)	*k* = −24→36
*T*_min_ = 0.919, *T*_max_ = 1.000	*l* = −21→21
52592 measured reflections	

### Sodium thiosulfate dihydrate (Na2S2O3H2O2_100K) Refinement

**Table:**

Refinement on *F*^2^	Primary atom site location: structure-invariant direct methods
Least-squares matrix: full	Secondary atom site location: difference Fourier map
*R*[*F*^2^ > 2σ(*F*^2^)] = 0.017	Hydrogen site location: difference Fourier map
*wR*(*F*^2^) = 0.047	All H-atom parameters refined
*S* = 1.02	*w* = 1/[σ^2^(*F*_o_^2^) + (0.0285*P*)^2^ + 0.0499*P*] where *P* = (*F*_o_^2^ + 2*F*_c_^2^)/3
8735 reflections	(Δ/σ)_max_ = 0.002
195 parameters	Δρ_max_ = 0.55 e Å^−^^3^
0 restraints	Δρ_min_ = −0.41 e Å^−^^3^

### Sodium thiosulfate dihydrate (Na2S2O3H2O2_100K) Special details

**Table:**

Geometry. All esds (except the esd in the dihedral angle between two l.s. planes) are estimated using the full covariance matrix. The cell esds are taken into account individually in the estimation of esds in distances, angles and torsion angles; correlations between esds in cell parameters are only used when they are defined by crystal symmetry. An approximate (isotropic) treatment of cell esds is used for estimating esds involving l.s. planes.

### Sodium thiosulfate dihydrate (Na2S2O3H2O2_100K) Fractional atomic coordinates and isotropic or equivalent isotropic displacement parameters (Å^2^)

**Table:**

	*x*	*y*	*z*	*U*_iso_*/*U*_eq_	
Na1	0.76421 (4)	0.06060 (2)	0.07970 (2)	0.01045 (4)	
Na2	0.77035 (4)	0.05630 (2)	0.42885 (2)	0.01123 (4)	
Na3	0.69690 (4)	0.18045 (2)	0.68402 (2)	0.00931 (4)	
Na4	0.25074 (4)	0.16699 (2)	0.96445 (2)	0.01068 (4)	
S1	0.40448 (2)	0.21188 (2)	0.40032 (2)	0.00580 (2)	
S2	0.05139 (2)	0.22163 (2)	0.34974 (2)	0.00789 (2)	
O1	0.46625 (7)	0.13787 (2)	0.40401 (4)	0.01077 (6)	
O2	0.48663 (6)	0.24383 (2)	0.51923 (3)	0.00914 (5)	
O3	0.51216 (6)	0.24942 (2)	0.31289 (3)	0.00948 (5)	
S3	0.50073 (2)	0.04913 (2)	0.80268 (2)	0.00634 (2)	
S4	0.85520 (2)	0.05914 (2)	0.83904 (2)	0.00764 (2)	
O4	0.40222 (7)	0.09813 (2)	0.70691 (4)	0.01385 (7)	
O5	0.41409 (7)	0.06361 (2)	0.91219 (3)	0.01001 (6)	
O6	0.43732 (7)	−0.02303 (2)	0.76485 (3)	0.01022 (6)	
O7	0.62228 (7)	0.17816 (2)	0.10472 (4)	0.01099 (6)	
H1	0.721 (2)	0.2050 (6)	0.0833 (10)	0.020 (2)*	
H2	0.610 (2)	0.1913 (6)	0.1672 (11)	0.024 (3)*	
O8	0.14212 (7)	0.11545 (2)	0.13791 (4)	0.01073 (6)	
H3	0.250 (2)	0.0899 (6)	0.1722 (10)	0.022 (3)*	
H4	0.117 (2)	0.1420 (8)	0.1836 (12)	0.036 (3)*	
O9	0.16296 (7)	0.02476 (2)	0.41193 (4)	0.01152 (6)	
H5	0.249 (3)	0.0597 (8)	0.4053 (13)	0.040 (4)*	
H6	0.162 (2)	0.0032 (7)	0.3500 (11)	0.028 (3)*	
O10	0.96285 (7)	0.13736 (2)	0.57734 (4)	0.01076 (6)	
H7	1.090 (2)	0.1261 (7)	0.6218 (11)	0.028 (3)*	
H8	0.987 (2)	0.1637 (7)	0.5266 (11)	0.030 (3)*	

### Sodium thiosulfate dihydrate (Na2S2O3H2O2_100K) Atomic displacement parameters (Å^2^)

**Table:**

	*U*^11^	*U*^22^	*U*^33^	*U*^12^	*U*^13^	*U*^23^
Na1	0.01049 (8)	0.01158 (9)	0.00939 (9)	−0.00200 (7)	0.00237 (7)	0.00018 (7)
Na2	0.01055 (9)	0.01247 (9)	0.00991 (9)	0.00314 (7)	0.00051 (7)	−0.00119 (7)
Na3	0.00991 (8)	0.00920 (8)	0.00892 (8)	0.00016 (6)	0.00226 (6)	0.00054 (6)
Na4	0.01100 (8)	0.00982 (9)	0.01091 (9)	0.00286 (7)	0.00168 (7)	−0.00021 (7)
S1	0.00541 (4)	0.00571 (4)	0.00618 (4)	0.00005 (3)	0.00100 (3)	−0.00008 (3)
S2	0.00558 (4)	0.01003 (4)	0.00789 (4)	0.00030 (3)	0.00106 (3)	0.00014 (3)
O1	0.00952 (13)	0.00573 (13)	0.01647 (16)	0.00125 (10)	0.00145 (11)	−0.00059 (11)
O2	0.00966 (13)	0.01030 (14)	0.00666 (12)	−0.00023 (10)	−0.00002 (10)	−0.00154 (10)
O3	0.00852 (12)	0.01143 (14)	0.00930 (13)	−0.00052 (10)	0.00372 (10)	0.00192 (10)
S3	0.00611 (4)	0.00644 (4)	0.00640 (4)	−0.00036 (3)	0.00113 (3)	0.00010 (3)
S4	0.00626 (4)	0.00816 (4)	0.00842 (4)	−0.00021 (3)	0.00138 (3)	0.00005 (3)
O4	0.00915 (13)	0.01576 (16)	0.01486 (16)	−0.00036 (12)	−0.00138 (12)	0.00848 (13)
O5	0.00940 (13)	0.01171 (14)	0.00994 (14)	0.00015 (11)	0.00431 (11)	−0.00291 (11)
O6	0.01128 (13)	0.00868 (13)	0.01099 (14)	−0.00318 (10)	0.00305 (11)	−0.00345 (10)
O7	0.01116 (14)	0.01221 (15)	0.00987 (14)	−0.00230 (11)	0.00287 (11)	−0.00060 (11)
O8	0.01084 (14)	0.00978 (14)	0.01119 (14)	0.00123 (11)	0.00152 (11)	−0.00083 (11)
O9	0.01357 (15)	0.01038 (14)	0.01172 (15)	−0.00148 (11)	0.00516 (12)	−0.00135 (11)
O10	0.00916 (13)	0.01293 (15)	0.01009 (14)	0.00096 (11)	0.00183 (11)	0.00219 (11)

### Sodium thiosulfate dihydrate (Na2S2O3H2O2_100K) Geometric parameters (Å, º)

**Table:**

Na1—O8^i^	2.3882 (5)	Na4—Na3^x^	3.9395 (3)
Na1—O6^ii^	2.4451 (5)	Na4—Na3^xi^	4.0357 (3)
Na1—O7	2.4529 (5)	S1—O1	1.4725 (4)
Na1—O5^iii^	2.4771 (5)	S1—O3	1.4815 (4)
Na1—O5^ii^	2.6214 (5)	S1—O2	1.4841 (4)
Na1—S4^iii^	2.9296 (3)	S1—S2	2.0047 (2)
Na1—S3^ii^	3.0917 (3)	S1—Na4^viii^	3.0636 (3)
Na1—S4^iv^	3.1898 (3)	S1—Na3^xiii^	3.2694 (3)
Na1—S3^iii^	3.2338 (3)	S2—Na3^xiii^	2.9354 (3)
Na1—Na4^iii^	3.6173 (3)	S2—Na4^xiii^	3.2208 (3)
Na1—Na4^v^	3.9337 (3)	O2—Na4^viii^	2.4708 (4)
Na1—Na1^vi^	3.9694 (5)	O3—Na3^xiii^	2.4883 (4)
Na2—O1	2.3307 (4)	O3—Na4^viii^	2.5536 (4)
Na2—O9^ii^	2.3794 (5)	S3—O4	1.4722 (4)
Na2—O6^ii^	2.3831 (4)	S3—O5	1.4797 (4)
Na2—O9^i^	2.3947 (5)	S3—O6	1.4832 (4)
Na2—O10	2.4078 (5)	S3—S4	2.0078 (2)
Na2—Na2^iv^	3.5475 (5)	S3—Na1^ii^	3.0917 (3)
Na2—Na3	3.8866 (3)	S3—Na1^ix^	3.2338 (3)
Na2—H8	2.557 (12)	S4—Na1^ix^	2.9295 (3)
Na3—O10	2.3169 (5)	S4—Na1^iv^	3.1897 (3)
Na3—O2	2.3636 (4)	S4—Na4^i^	3.1981 (3)
Na3—O4	2.3845 (5)	O5—Na1^ix^	2.4771 (5)
Na3—O3^vii^	2.4884 (4)	O5—Na1^ii^	2.6213 (5)
Na3—S2^vii^	2.9354 (3)	O6—Na2^ii^	2.3831 (4)
Na3—S4	2.9695 (3)	O6—Na1^ii^	2.4451 (5)
Na3—S3	3.2022 (3)	O7—Na4^iii^	2.4018 (5)
Na3—S1^vii^	3.2694 (3)	O7—H1	0.844 (11)
Na3—Na4^viii^	3.9395 (3)	O7—H2	0.782 (12)
Na3—Na4^i^	4.0357 (3)	O8—Na1^xi^	2.3882 (5)
Na4—O5	2.3423 (4)	O8—Na4^iii^	2.4316 (5)
Na4—O7^ix^	2.4017 (5)	O8—H3	0.827 (12)
Na4—O8^ix^	2.4317 (5)	O8—H4	0.772 (14)
Na4—O2^x^	2.4708 (4)	O9—Na2^ii^	2.3793 (5)
Na4—O3^x^	2.5536 (4)	O9—Na2^xi^	2.3947 (5)
Na4—S1^x^	3.0636 (2)	O9—H5	0.851 (15)
Na4—S4^xi^	3.1981 (3)	O9—H6	0.825 (13)
Na4—S2^vii^	3.2208 (3)	O10—H7	0.828 (13)
Na4—Na1^ix^	3.6173 (3)	O10—H8	0.810 (13)
Na4—Na1^xii^	3.9337 (3)		
			
O8^i^—Na1—O6^ii^	118.400 (16)	O5—Na4—O7^ix^	84.197 (15)
O8^i^—Na1—O7	82.178 (15)	O5—Na4—O8^ix^	92.899 (16)
O6^ii^—Na1—O7	88.032 (15)	O7^ix^—Na4—O8^ix^	80.483 (15)
O8^i^—Na1—O5^iii^	138.729 (17)	O5—Na4—O2^x^	164.794 (17)
O6^ii^—Na1—O5^iii^	98.068 (15)	O7^ix^—Na4—O2^x^	106.048 (16)
O7—Na1—O5^iii^	80.357 (15)	O8^ix^—Na4—O2^x^	78.118 (14)
O8^i^—Na1—O5^ii^	137.591 (16)	O5—Na4—O3^x^	123.483 (16)
O6^ii^—Na1—O5^ii^	56.628 (13)	O7^ix^—Na4—O3^x^	132.507 (17)
O7—Na1—O5^ii^	134.460 (16)	O8^ix^—Na4—O3^x^	128.636 (15)
O5^iii^—Na1—O5^ii^	77.780 (15)	O2^x^—Na4—O3^x^	57.417 (12)
O8^i^—Na1—S4^iii^	86.073 (12)	O5—Na4—S1^x^	150.169 (14)
O6^ii^—Na1—S4^iii^	154.130 (13)	O7^ix^—Na4—S1^x^	122.393 (13)
O7—Na1—S4^iii^	104.610 (12)	O8^ix^—Na4—S1^x^	103.959 (12)
O5^iii^—Na1—S4^iii^	62.959 (10)	O2^x^—Na4—S1^x^	28.631 (9)
O5^ii^—Na1—S4^iii^	100.155 (11)	O3^x^—Na4—S1^x^	28.794 (9)
O8^i^—Na1—S3^ii^	133.571 (13)	O5—Na4—S4^xi^	67.401 (11)
O6^ii^—Na1—S3^ii^	28.098 (9)	O7^ix^—Na4—S4^xi^	144.113 (14)
O7—Na1—S3^ii^	112.008 (13)	O8^ix^—Na4—S4^xi^	79.579 (12)
O5^iii^—Na1—S3^ii^	87.693 (11)	O2^x^—Na4—S4^xi^	98.634 (12)
O5^ii^—Na1—S3^ii^	28.531 (9)	O3^x^—Na4—S4^xi^	82.850 (11)
S4^iii^—Na1—S3^ii^	127.864 (9)	S1^x^—Na4—S4^xi^	91.346 (7)
O8^i^—Na1—S4^iv^	73.118 (12)	O5—Na4—S2^vii^	100.525 (12)
O6^ii^—Na1—S4^iv^	88.603 (12)	O7^ix^—Na4—S2^vii^	74.571 (12)
O7—Na1—S4^iv^	149.747 (13)	O8^ix^—Na4—S2^vii^	150.145 (13)
O5^iii^—Na1—S4^iv^	129.865 (13)	O2^x^—Na4—S2^vii^	93.140 (11)
O5^ii^—Na1—S4^iv^	64.918 (10)	O3^x^—Na4—S2^vii^	63.633 (10)
S4^iii^—Na1—S4^iv^	91.068 (7)	S1^x^—Na4—S2^vii^	76.791 (6)
S3^ii^—Na1—S4^iv^	75.409 (6)	S4^xi^—Na4—S2^vii^	130.207 (8)
O8^i^—Na1—S3^iii^	121.015 (13)	O5—Na4—Na1^ix^	42.791 (11)
O6^ii^—Na1—S3^iii^	120.330 (12)	O7^ix^—Na4—Na1^ix^	42.373 (11)
O7—Na1—S3^iii^	94.657 (12)	O8^ix^—Na4—Na1^ix^	78.527 (12)
O5^iii^—Na1—S3^iii^	25.963 (9)	O2^x^—Na4—Na1^ix^	143.576 (12)
O5^ii^—Na1—S3^iii^	82.368 (11)	O3^x^—Na4—Na1^ix^	152.820 (13)
S4^iii^—Na1—S3^iii^	37.616 (4)	S1^x^—Na4—Na1^ix^	164.477 (9)
S3^ii^—Na1—S3^iii^	102.300 (7)	S4^xi^—Na4—Na1^ix^	104.156 (8)
S4^iv^—Na1—S3^iii^	112.843 (8)	S2^vii^—Na4—Na1^ix^	93.397 (7)
O8^i^—Na1—Na4^iii^	118.543 (13)	O5—Na4—Na1^xii^	89.810 (12)
O6^ii^—Na1—Na4^iii^	87.500 (12)	O7^ix^—Na4—Na1^xii^	114.775 (13)
O7—Na1—Na4^iii^	41.293 (11)	O8^ix^—Na4—Na1^xii^	34.934 (10)
O5^iii^—Na1—Na4^iii^	39.967 (10)	O2^x^—Na4—Na1^xii^	75.773 (11)
O5^ii^—Na1—Na4^iii^	103.685 (11)	O3^x^—Na4—Na1^xii^	104.000 (11)
S4^iii^—Na1—Na4^iii^	87.559 (7)	S1^x^—Na4—Na1^xii^	90.270 (7)
S3^ii^—Na1—Na4^iii^	96.159 (8)	S4^xi^—Na4—Na1^xii^	47.125 (5)
S4^iv^—Na1—Na4^iii^	168.094 (9)	S2^vii^—Na4—Na1^xii^	166.945 (8)
S3^iii^—Na1—Na4^iii^	60.141 (6)	Na1^ix^—Na4—Na1^xii^	99.620 (8)
O8^i^—Na1—Na4^v^	35.666 (11)	O5—Na4—Na3^x^	153.485 (13)
O6^ii^—Na1—Na4^v^	152.675 (13)	O7^ix^—Na4—Na3^x^	71.525 (12)
O7—Na1—Na4^v^	80.568 (11)	O8^ix^—Na4—Na3^x^	73.117 (12)
O5^iii^—Na1—Na4^v^	104.310 (12)	O2^x^—Na4—Na3^x^	34.529 (10)
O5^ii^—Na1—Na4^v^	143.591 (12)	O3^x^—Na4—Na3^x^	81.957 (11)
S4^iii^—Na1—Na4^v^	53.130 (5)	S1^x^—Na4—Na3^x^	56.340 (6)
S3^ii^—Na1—Na4^v^	164.219 (9)	S4^xi^—Na4—Na3^x^	129.098 (8)
S4^iv^—Na1—Na4^v^	88.948 (7)	S2^vii^—Na4—Na3^x^	83.498 (7)
S3^iii^—Na1—Na4^v^	85.509 (7)	Na1^ix^—Na4—Na3^x^	111.170 (8)
Na4^iii^—Na1—Na4^v^	99.621 (8)	Na1^xii^—Na4—Na3^x^	90.819 (7)
O8^i^—Na1—Na1^vi^	163.063 (16)	O5—Na4—Na3^xi^	98.206 (12)
O6^ii^—Na1—Na1^vi^	73.997 (11)	O7^ix^—Na4—Na3^xi^	166.439 (14)
O7—Na1—Na1^vi^	110.978 (14)	O8^ix^—Na4—Na3^xi^	112.594 (12)
O5^iii^—Na1—Na1^vi^	40.198 (10)	O2^x^—Na4—Na3^xi^	74.523 (10)
O5^ii^—Na1—Na1^vi^	37.582 (9)	O3^x^—Na4—Na3^xi^	36.277 (10)
S4^iii^—Na1—Na1^vi^	80.371 (8)	S1^x^—Na4—Na3^xi^	52.703 (5)
S3^ii^—Na1—Na1^vi^	52.748 (6)	S4^xi^—Na4—Na3^xi^	46.719 (5)
S4^iv^—Na1—Na1^vi^	96.895 (9)	S2^vii^—Na4—Na3^xi^	91.873 (7)
S3^iii^—Na1—Na1^vi^	49.552 (6)	Na1^ix^—Na4—Na3^xi^	140.915 (8)
Na4^iii^—Na1—Na1^vi^	71.213 (8)	Na1^xii^—Na4—Na3^xi^	78.663 (6)
Na4^v^—Na1—Na1^vi^	133.293 (10)	Na3^x^—Na4—Na3^xi^	107.902 (7)
O1—Na2—O9^ii^	122.205 (18)	O1—S1—O3	111.16 (2)
O1—Na2—O6^ii^	81.562 (16)	O1—S1—O2	110.48 (2)
O9^ii^—Na2—O6^ii^	120.629 (17)	O3—S1—O2	109.02 (2)
O1—Na2—O9^i^	149.663 (18)	O1—S1—S2	108.927 (16)
O9^ii^—Na2—O9^i^	84.012 (16)	O3—S1—S2	107.764 (16)
O6^ii^—Na2—O9^i^	98.619 (16)	O2—S1—S2	109.430 (16)
O1—Na2—O10	82.399 (15)	O1—S1—Na4^viii^	126.430 (16)
O9^ii^—Na2—O10	84.685 (15)	O3—S1—Na4^viii^	56.126 (16)
O6^ii^—Na2—O10	154.570 (17)	O2—S1—Na4^viii^	52.913 (16)
O9^i^—Na2—O10	85.577 (16)	S2—S1—Na4^viii^	124.628 (7)
O1—Na2—Na2^iv^	160.062 (17)	O1—S1—Na3^xiii^	133.400 (18)
O9^ii^—Na2—Na2^iv^	42.172 (11)	O3—S1—Na3^xiii^	46.304 (15)
O6^ii^—Na2—Na2^iv^	116.288 (15)	O2—S1—Na3^xiii^	115.505 (16)
O9^i^—Na2—Na2^iv^	41.840 (11)	S2—S1—Na3^xiii^	62.309 (6)
O10—Na2—Na2^iv^	83.443 (13)	Na4^viii^—S1—Na3^xiii^	79.101 (7)
O1—Na2—Na3	58.255 (12)	S1—S2—Na3^xiii^	80.481 (7)
O9^ii^—Na2—Na3	81.351 (12)	S1—S2—Na4^xiii^	123.174 (7)
O6^ii^—Na2—Na3	139.441 (13)	Na3^xiii^—S2—Na4^xiii^	95.180 (7)
O9^i^—Na2—Na3	118.510 (13)	S1—O1—Na2	146.29 (2)
O10—Na2—Na3	33.899 (10)	S1—O2—Na3	122.18 (2)
Na2^iv^—Na2—Na3	102.788 (10)	S1—O2—Na4^viii^	98.46 (2)
O1—Na2—Na1	91.058 (13)	Na3—O2—Na4^viii^	109.136 (16)
O9^ii^—Na2—Na1	138.654 (13)	S1—O3—Na3^xiii^	108.20 (2)
O6^ii^—Na2—Na1	34.248 (10)	S1—O3—Na4^viii^	95.079 (19)
O9^i^—Na2—Na1	74.270 (12)	Na3^xiii^—O3—Na4^viii^	106.335 (16)
O10—Na2—Na1	126.973 (13)	O4—S3—O5	111.71 (2)
Na2^iv^—Na2—Na1	108.694 (10)	O4—S3—O6	110.70 (2)
Na3—Na2—Na1	139.958 (8)	O5—S3—O6	108.72 (2)
O1—Na2—H8	77.9 (3)	O4—S3—S4	107.862 (17)
O9^ii^—Na2—H8	102.3 (3)	O5—S3—S4	108.640 (16)
O6^ii^—Na2—H8	137.0 (3)	O6—S3—S4	109.151 (17)
O9^i^—Na2—H8	82.0 (3)	O4—S3—Na1^ii^	128.431 (17)
O10—Na2—H8	18.5 (3)	O5—S3—Na1^ii^	57.791 (16)
Na2^iv^—Na2—H8	92.7 (3)	O6—S3—Na1^ii^	50.932 (16)
Na3—Na2—H8	44.6 (3)	S4—S3—Na1^ii^	123.533 (7)
Na1—Na2—H8	108.8 (3)	O4—S3—Na3	44.406 (17)
O10—Na3—O2	92.578 (16)	O5—S3—Na3	115.395 (17)
O10—Na3—O4	112.849 (18)	O6—S3—Na3	135.020 (17)
O2—Na3—O4	100.309 (15)	S4—S3—Na3	64.837 (6)
O10—Na3—O3^vii^	91.667 (15)	Na1^ii^—S3—Na3	169.724 (7)
O2—Na3—O3^vii^	112.361 (16)	O4—S3—Na1^ix^	135.99 (2)
O4—Na3—O3^vii^	138.171 (17)	O5—S3—Na1^ix^	47.128 (16)
O10—Na3—S2^vii^	152.992 (14)	O6—S3—Na1^ix^	112.854 (17)
O2—Na3—S2^vii^	91.069 (12)	S4—S3—Na1^ix^	62.944 (6)
O4—Na3—S2^vii^	92.745 (14)	Na1^ii^—S3—Na1^ix^	77.699 (7)
O3^vii^—Na3—S2^vii^	62.335 (10)	Na3—S3—Na1^ix^	103.422 (7)
O10—Na3—S4	83.204 (12)	S3—S4—Na1^ix^	79.440 (7)
O2—Na3—S4	158.528 (13)	S3—S4—Na3	77.431 (6)
O4—Na3—S4	62.715 (11)	Na1^ix^—S4—Na3	117.818 (8)
O3^vii^—Na3—S4	88.861 (11)	S3—S4—Na1^iv^	126.733 (7)
S2^vii^—Na3—S4	102.249 (8)	Na1^ix^—S4—Na1^iv^	88.932 (7)
O10—Na3—S3	106.058 (13)	Na3—S4—Na1^iv^	148.524 (7)
O2—Na3—S3	125.851 (13)	S3—S4—Na4^i^	138.919 (7)
O4—Na3—S3	25.595 (10)	Na1^ix^—S4—Na4^i^	79.744 (7)
O3^vii^—Na3—S3	117.222 (12)	Na3—S4—Na4^i^	81.648 (7)
S2^vii^—Na3—S3	93.258 (7)	Na1^iv^—S4—Na4^i^	87.727 (7)
S4—Na3—S3	37.733 (4)	S3—O4—Na3	110.00 (2)
O10—Na3—S1^vii^	117.162 (13)	S3—O5—Na4	127.49 (2)
O2—Na3—S1^vii^	108.846 (12)	S3—O5—Na1^ix^	106.91 (2)
O4—Na3—S1^vii^	119.596 (14)	Na4—O5—Na1^ix^	97.241 (16)
O3^vii^—Na3—S1^vii^	25.496 (9)	S3—O5—Na1^ii^	93.680 (19)
S2^vii^—Na3—S1^vii^	37.209 (4)	Na4—O5—Na1^ii^	126.101 (17)
S4—Na3—S1^vii^	91.674 (7)	Na1^ix^—O5—Na1^ii^	102.221 (15)
S3—Na3—S1^vii^	106.535 (7)	S3—O6—Na2^ii^	124.85 (2)
O10—Na3—Na2	35.423 (11)	S3—O6—Na1^ii^	100.97 (2)
O2—Na3—Na2	80.408 (11)	Na2^ii^—O6—Na1^ii^	112.489 (17)
O4—Na3—Na2	82.405 (14)	Na4^iii^—O7—Na1	96.333 (16)
O3^vii^—Na3—Na2	127.067 (12)	Na4^iii^—O7—H1	114.7 (8)
S2^vii^—Na3—Na2	169.225 (9)	Na1—O7—H1	105.9 (8)
S4—Na3—Na2	84.133 (7)	Na4^iii^—O7—H2	113.7 (9)
S3—Na3—Na2	86.521 (7)	Na1—O7—H2	120.6 (9)
S1^vii^—Na3—Na2	152.539 (8)	H1—O7—H2	105.7 (11)
O10—Na3—Na4^viii^	77.064 (12)	Na1^xi^—O8—Na4^iii^	109.400 (17)
O2—Na3—Na4^viii^	36.336 (10)	Na1^xi^—O8—H3	114.8 (8)
O4—Na3—Na4^viii^	136.606 (12)	Na4^iii^—O8—H3	109.6 (8)
O3^vii^—Na3—Na4^viii^	80.031 (11)	Na1^xi^—O8—H4	100.9 (10)
S2^vii^—Na3—Na4^viii^	90.445 (7)	Na4^iii^—O8—H4	114.1 (10)
S4—Na3—Na4^viii^	156.961 (8)	H3—O8—H4	107.9 (13)
S3—Na3—Na4^viii^	161.959 (8)	Na2^ii^—O9—Na2^xi^	95.988 (16)
S1^vii^—Na3—Na4^viii^	86.918 (7)	Na2^ii^—O9—H5	126.2 (10)
Na2—Na3—Na4^viii^	86.578 (7)	Na2^xi^—O9—H5	112.6 (10)
O10—Na3—Na4^i^	84.262 (12)	Na2^ii^—O9—H6	107.7 (9)
O2—Na3—Na4^i^	149.099 (13)	Na2^xi^—O9—H6	111.6 (9)
O4—Na3—Na4^i^	109.260 (12)	H5—O9—H6	102.7 (12)
O3^vii^—Na3—Na4^i^	37.389 (10)	Na3—O10—Na2	110.678 (17)
S2^vii^—Na3—Na4^i^	79.004 (6)	Na3—O10—H7	111.4 (8)
S4—Na3—Na4^i^	51.632 (5)	Na2—O10—H7	118.7 (9)
S3—Na3—Na4^i^	84.222 (6)	Na3—O10—H8	113.2 (9)
S1^vii^—Na3—Na4^i^	48.197 (5)	Na2—O10—H8	91.3 (9)
Na2—Na3—Na4^i^	111.661 (7)	H7—O10—H8	110.2 (12)
Na4^viii^—Na3—Na4^i^	113.819 (7)		

Symmetry codes: (i) *x*+1, *y*, *z*; (ii) −*x*+1, −*y*, −*z*+1; (iii) *x*, *y*, *z*−1; (iv) −*x*+2, −*y*, −*z*+1; (v) *x*+1, *y*, *z*−1; (vi) −*x*+1, −*y*, −*z*; (vii) *x*+1/2, −*y*+1/2, *z*+1/2; (viii) *x*+1/2, −*y*+1/2, *z*−1/2; (ix) *x*, *y*, *z*+1; (x) *x*−1/2, −*y*+1/2, *z*+1/2; (xi) *x*−1, *y*, *z*; (xii) *x*−1, *y*, *z*+1; (xiii) *x*−1/2, −*y*+1/2, *z*−1/2.

### Sodium thiosulfate dihydrate (Na2S2O3H2O2_100K) Hydrogen-bond geometry (Å, º)

**Table:**

*D*—H···*A*	*D*—H	H···*A*	*D*···*A*	*D*—H···*A*
O7—H1···O2^viii^	0.844 (11)	2.090 (11)	2.9246 (5)	170.1 (11)
O7—H2···O3	0.782 (12)	2.190 (12)	2.9498 (6)	164.3 (12)
O8—H3···O6^ii^	0.827 (12)	2.211 (12)	3.0282 (6)	170.1 (11)
O8—H4···S2	0.772 (14)	2.545 (14)	3.3142 (4)	174.5 (13)
O9—H5···O1	0.851 (15)	1.966 (15)	2.8142 (6)	174.7 (15)
O9—H6···S4^ii^	0.825 (13)	2.471 (13)	3.2959 (4)	177.9 (12)
O10—H7···O4^i^	0.828 (13)	1.936 (13)	2.7585 (5)	172.4 (12)
O10—H8···S2^i^	0.810 (13)	2.421 (13)	3.2183 (4)	168.3 (12)

Symmetry codes: (i) *x*+1, *y*, *z*; (ii) −*x*+1, −*y*, −*z*+1; (viii) *x*+1/2, −*y*+1/2, *z*−1/2.

### (Na2S2O3H2O2_200K) Crystal data

**Table:**

O_3_S_2_·2(H_2_O)·2(Na)	*F*(000) = 784
*M**_r_* = 194.13	*D*_x_ = 2.034 Mg m^−^^3^
Monoclinic, *P*2_1_/*n*	Mo *K*α radiation, λ = 0.71073 Å
*a* = 5.8003 (1) Å	Cell parameters from 63400 reflections
*b* = 19.3713 (4) Å	θ = 3.2–42.5°
*c* = 11.5520 (3) Å	µ = 0.92 mm^−^^1^
β = 102.331 (2)°	*T* = 200 K
*V* = 1268.03 (5) Å^3^	Block, colourless
*Z* = 8	0.25 × 0.2 × 0.15 mm

### (Na2S2O3H2O2_200K) Data collection

**Table:**

Stoe StadiVari diffractometer	8848 independent reflections
Radiation source: Genix 3D HF Mo	7181 reflections with *I* > 2σ(*I*)
Graded multilayer mirror monochromator	*R*_int_ = 0.024
Detector resolution: 5.81 pixels mm^-1^	θ_max_ = 42.3°, θ_min_ = 3.6°
ω scans	*h* = −10→10
Absorption correction: empirical (using intensity measurements) (X-AREA; Stoe & Cie, 2015)	*k* = −24→36
*T*_min_ = 0.920, *T*_max_ = 1.000	*l* = −21→21
55208 measured reflections	

### (Na2S2O3H2O2_200K) Refinement

**Table:**

Refinement on *F*^2^	Primary atom site location: structure-invariant direct methods
Least-squares matrix: full	Secondary atom site location: difference Fourier map
*R*[*F*^2^ > 2σ(*F*^2^)] = 0.022	Hydrogen site location: difference Fourier map
*wR*(*F*^2^) = 0.057	All H-atom parameters refined
*S* = 1.01	*w* = 1/[σ^2^(*F*_o_^2^) + (0.0343*P*)^2^] where *P* = (*F*_o_^2^ + 2*F*_c_^2^)/3
8848 reflections	(Δ/σ)_max_ = 0.002
195 parameters	Δρ_max_ = 0.58 e Å^−^^3^
0 restraints	Δρ_min_ = −0.34 e Å^−^^3^

### (Na2S2O3H2O2_200K) Special details

**Table:**

Geometry. All esds (except the esd in the dihedral angle between two l.s. planes) are estimated using the full covariance matrix. The cell esds are taken into account individually in the estimation of esds in distances, angles and torsion angles; correlations between esds in cell parameters are only used when they are defined by crystal symmetry. An approximate (isotropic) treatment of cell esds is used for estimating esds involving l.s. planes.

### (Na2S2O3H2O2_200K) Fractional atomic coordinates and isotropic or equivalent isotropic displacement parameters (Å^2^)

**Table:**

	*x*	*y*	*z*	*U*_iso_*/*U*_eq_	
Na1	0.76083 (5)	0.06118 (2)	0.07884 (3)	0.01992 (5)	
Na2	0.77256 (5)	0.05700 (2)	0.42842 (3)	0.02061 (5)	
Na3	0.69341 (5)	0.18066 (2)	0.68350 (2)	0.01663 (5)	
Na4	0.24774 (5)	0.16710 (2)	0.96288 (3)	0.01997 (5)	
S1	0.40321 (2)	0.21139 (2)	0.39998 (2)	0.01053 (2)	
S2	0.05175 (2)	0.22031 (2)	0.34932 (2)	0.01460 (3)	
O1	0.46682 (8)	0.13784 (2)	0.40371 (5)	0.01990 (8)	
O2	0.48340 (8)	0.24334 (2)	0.51827 (4)	0.01645 (7)	
O3	0.50946 (8)	0.24916 (3)	0.31300 (4)	0.01705 (7)	
S3	0.49533 (2)	0.04958 (2)	0.80242 (2)	0.01214 (3)	
S4	0.84805 (2)	0.05883 (2)	0.83802 (2)	0.01430 (3)	
O4	0.39836 (9)	0.09907 (3)	0.70831 (5)	0.02776 (11)	
O5	0.41052 (8)	0.06351 (3)	0.91195 (4)	0.01854 (8)	
O6	0.43089 (9)	−0.02198 (3)	0.76389 (4)	0.02031 (8)	
O7	0.61781 (9)	0.17868 (3)	0.10362 (4)	0.01977 (8)	
H1	0.714 (2)	0.2063 (6)	0.0822 (12)	0.032 (3)*	
H2	0.603 (2)	0.1921 (7)	0.1666 (12)	0.034 (3)*	
O8	0.13817 (9)	0.11508 (3)	0.13587 (5)	0.01947 (8)	
H3	0.242 (2)	0.0895 (7)	0.1678 (11)	0.032 (3)*	
H4	0.116 (3)	0.1393 (9)	0.1796 (14)	0.055 (4)*	
O9	0.16529 (10)	0.02449 (3)	0.41317 (5)	0.02058 (8)	
H5	0.248 (3)	0.0584 (9)	0.4037 (16)	0.059 (5)*	
H6	0.165 (2)	0.0039 (7)	0.3523 (12)	0.033 (3)*	
O10	0.96144 (8)	0.13779 (3)	0.57813 (4)	0.01861 (8)	
H7	1.088 (2)	0.1259 (7)	0.6229 (12)	0.038 (3)*	
H8	0.990 (2)	0.1628 (7)	0.5310 (12)	0.037 (3)*	

### (Na2S2O3H2O2_200K) Atomic displacement parameters (Å^2^)

**Table:**

	*U*^11^	*U*^22^	*U*^33^	*U*^12^	*U*^13^	*U*^23^
Na1	0.02111 (12)	0.02219 (13)	0.01677 (11)	−0.00539 (9)	0.00473 (9)	−0.00005 (9)
Na2	0.01952 (12)	0.02307 (13)	0.01777 (12)	0.00628 (9)	0.00067 (9)	−0.00274 (9)
Na3	0.01804 (11)	0.01634 (11)	0.01560 (11)	0.00029 (8)	0.00381 (8)	0.00127 (8)
Na4	0.02085 (12)	0.01800 (12)	0.02026 (12)	0.00594 (9)	0.00261 (9)	−0.00061 (9)
S1	0.01007 (4)	0.01019 (5)	0.01107 (5)	0.00009 (3)	0.00164 (3)	−0.00023 (3)
S2	0.01013 (5)	0.01901 (6)	0.01424 (5)	0.00055 (4)	0.00167 (4)	0.00046 (4)
O1	0.01663 (17)	0.01085 (17)	0.0309 (2)	0.00213 (13)	0.00209 (16)	−0.00127 (15)
O2	0.01729 (17)	0.01861 (18)	0.01175 (16)	−0.00063 (14)	−0.00069 (13)	−0.00254 (13)
O3	0.01504 (16)	0.02101 (19)	0.01647 (18)	−0.00113 (14)	0.00641 (13)	0.00321 (14)
S3	0.01239 (5)	0.01208 (5)	0.01166 (5)	−0.00133 (4)	0.00192 (4)	0.00020 (4)
S4	0.01222 (5)	0.01537 (6)	0.01526 (6)	0.00022 (4)	0.00283 (4)	0.00047 (4)
O4	0.01646 (19)	0.0326 (3)	0.0303 (3)	−0.00209 (18)	−0.00376 (17)	0.0184 (2)
O5	0.01686 (17)	0.0215 (2)	0.01898 (19)	0.00002 (14)	0.00763 (15)	−0.00642 (15)
O6	0.0240 (2)	0.01761 (19)	0.0204 (2)	−0.00826 (16)	0.00714 (16)	−0.00775 (15)
O7	0.02038 (19)	0.0217 (2)	0.01769 (19)	−0.00475 (16)	0.00510 (15)	−0.00113 (15)
O8	0.02040 (19)	0.01723 (19)	0.0201 (2)	0.00224 (15)	0.00281 (16)	−0.00136 (15)
O9	0.0244 (2)	0.0185 (2)	0.0211 (2)	−0.00272 (16)	0.00981 (17)	−0.00309 (16)
O10	0.01630 (18)	0.0226 (2)	0.01676 (18)	0.00175 (15)	0.00311 (14)	0.00375 (15)

### (Na2S2O3H2O2_200K) Geometric parameters (Å, º)

**Table:**

Na1—O8^i^	2.3873 (6)	Na4—Na3^x^	3.9539 (4)
Na1—O6^ii^	2.4447 (6)	Na4—Na3^xi^	4.0486 (4)
Na1—O7	2.4602 (6)	S1—O1	1.4702 (5)
Na1—O5^iii^	2.4848 (6)	S1—O3	1.4795 (5)
Na1—O5^ii^	2.6227 (6)	S1—O2	1.4816 (4)
Na1—S4^iii^	2.9323 (3)	S1—S2	2.0047 (2)
Na1—S3^ii^	3.0924 (3)	S1—Na4^viii^	3.0727 (3)
Na1—S3^iii^	3.2433 (3)	S1—Na3^xiii^	3.2875 (3)
Na1—S4^iv^	3.2470 (3)	S2—Na3^xiii^	2.9490 (3)
Na1—Na4^iii^	3.6275 (4)	S2—Na4^xiii^	3.2520 (3)
Na1—Na4^v^	3.9492 (4)	O2—Na4^viii^	2.4878 (5)
Na1—Na1^vi^	3.9670 (6)	O3—Na3^xiii^	2.5051 (5)
Na2—O1	2.3372 (5)	O3—Na4^viii^	2.5529 (6)
Na2—O6^ii^	2.3808 (6)	S3—O4	1.4684 (5)
Na2—O9^ii^	2.3851 (6)	S3—O5	1.4770 (5)
Na2—O9^i^	2.4058 (6)	S3—O6	1.4793 (5)
Na2—O10	2.4148 (6)	S3—S4	2.0068 (2)
Na2—Na2^iv^	3.5650 (6)	S3—Na1^ii^	3.0924 (3)
Na2—Na3	3.9000 (4)	S3—Na1^ix^	3.2432 (3)
Na2—H8	2.560 (13)	S4—Na1^ix^	2.9323 (3)
Na3—O10	2.3231 (6)	S4—Na4^i^	3.2288 (3)
Na3—O2	2.3681 (5)	S4—Na1^iv^	3.2470 (3)
Na3—O4	2.3914 (6)	O5—Na1^ix^	2.4848 (6)
Na3—O3^vii^	2.5051 (5)	O5—Na1^ii^	2.6227 (6)
Na3—S2^vii^	2.9491 (3)	O6—Na2^ii^	2.3808 (6)
Na3—S4	2.9794 (3)	O6—Na1^ii^	2.4447 (6)
Na3—S3	3.2139 (3)	O7—Na4^iii^	2.4106 (6)
Na3—S1^vii^	3.2874 (3)	O7—H1	0.847 (13)
Na3—Na4^viii^	3.9540 (4)	O7—H2	0.795 (13)
Na3—Na4^i^	4.0486 (4)	O8—Na1^xi^	2.3872 (6)
Na4—O5	2.3456 (5)	O8—Na4^iii^	2.4403 (6)
Na4—O7^ix^	2.4105 (6)	O8—H3	0.804 (13)
Na4—O8^ix^	2.4403 (6)	O8—H4	0.721 (17)
Na4—O2^x^	2.4877 (5)	O9—Na2^ii^	2.3851 (6)
Na4—O3^x^	2.5529 (6)	O9—Na2^xi^	2.4059 (6)
Na4—S1^x^	3.0726 (3)	O9—H5	0.835 (17)
Na4—S4^xi^	3.2289 (3)	O9—H6	0.808 (14)
Na4—S2^vii^	3.2520 (3)	O10—H7	0.835 (14)
Na4—Na1^ix^	3.6275 (4)	O10—H8	0.772 (14)
Na4—Na1^xii^	3.9493 (4)		
			
O8^i^—Na1—O6^ii^	117.81 (2)	O5—Na4—O7^ix^	84.30 (2)
O8^i^—Na1—O7	82.834 (19)	O5—Na4—O8^ix^	92.37 (2)
O6^ii^—Na1—O7	89.01 (2)	O7^ix^—Na4—O8^ix^	80.573 (19)
O8^i^—Na1—O5^iii^	139.09 (2)	O5—Na4—O2^x^	164.65 (2)
O6^ii^—Na1—O5^iii^	98.969 (19)	O7^ix^—Na4—O2^x^	105.701 (19)
O7—Na1—O5^iii^	80.403 (18)	O8^ix^—Na4—O2^x^	78.158 (18)
O8^i^—Na1—O5^ii^	136.44 (2)	O5—Na4—O3^x^	124.20 (2)
O6^ii^—Na1—O5^ii^	56.453 (16)	O7^ix^—Na4—O3^x^	132.33 (2)
O7—Na1—O5^ii^	135.01 (2)	O8^ix^—Na4—O3^x^	128.304 (19)
O5^iii^—Na1—O5^ii^	78.12 (2)	O2^x^—Na4—O3^x^	57.124 (15)
O8^i^—Na1—S4^iii^	86.263 (16)	O5—Na4—S1^x^	150.730 (17)
O6^ii^—Na1—S4^iii^	153.927 (16)	O7^ix^—Na4—S1^x^	121.920 (17)
O7—Na1—S4^iii^	104.804 (16)	O8^ix^—Na4—S1^x^	103.888 (16)
O5^iii^—Na1—S4^iii^	62.763 (12)	O2^x^—Na4—S1^x^	28.502 (10)
O5^ii^—Na1—S4^iii^	99.662 (14)	O3^x^—Na4—S1^x^	28.635 (10)
O8^i^—Na1—S3^ii^	132.852 (17)	O5—Na4—S4^xi^	67.748 (14)
O6^ii^—Na1—S3^ii^	27.992 (11)	O7^ix^—Na4—S4^xi^	144.363 (17)
O7—Na1—S3^ii^	112.607 (16)	O8^ix^—Na4—S4^xi^	79.041 (15)
O5^iii^—Na1—S3^ii^	88.056 (14)	O2^x^—Na4—S4^xi^	98.321 (14)
O5^ii^—Na1—S3^ii^	28.468 (11)	O3^x^—Na4—S4^xi^	82.917 (13)
S4^iii^—Na1—S3^ii^	127.348 (11)	S1^x^—Na4—S4^xi^	91.333 (8)
O8^i^—Na1—S3^iii^	121.104 (16)	O5—Na4—S2^vii^	100.986 (16)
O6^ii^—Na1—S3^iii^	120.962 (16)	O7^ix^—Na4—S2^vii^	74.230 (15)
O7—Na1—S3^iii^	94.456 (15)	O8^ix^—Na4—S2^vii^	149.970 (17)
O5^iii^—Na1—S3^iii^	25.800 (11)	O2^x^—Na4—S2^vii^	93.044 (14)
O5^ii^—Na1—S3^iii^	82.631 (13)	O3^x^—Na4—S2^vii^	64.017 (12)
S4^iii^—Na1—S3^iii^	37.501 (5)	S1^x^—Na4—S2^vii^	76.829 (8)
S3^ii^—Na1—S3^iii^	102.508 (9)	S4^xi^—Na4—S2^vii^	130.911 (10)
O8^i^—Na1—S4^iv^	71.943 (15)	O5—Na4—Na1^ix^	42.800 (14)
O6^ii^—Na1—S4^iv^	87.582 (16)	O7^ix^—Na4—Na1^ix^	42.386 (14)
O7—Na1—S4^iv^	149.277 (16)	O8^ix^—Na4—Na1^ix^	78.499 (15)
O5^iii^—Na1—S4^iv^	130.273 (16)	O2^x^—Na4—Na1^ix^	143.281 (15)
O5^ii^—Na1—S4^iv^	64.881 (13)	O3^x^—Na4—Na1^ix^	153.189 (16)
S4^iii^—Na1—S4^iv^	91.048 (9)	S1^x^—Na4—Na1^ix^	164.044 (11)
S3^ii^—Na1—S4^iv^	75.144 (8)	S4^xi^—Na4—Na1^ix^	104.579 (10)
S3^iii^—Na1—S4^iv^	113.356 (10)	S2^vii^—Na4—Na1^ix^	93.185 (9)
O8^i^—Na1—Na4^iii^	119.081 (17)	O5—Na4—Na1^xii^	89.768 (15)
O6^ii^—Na1—Na4^iii^	89.002 (17)	O7^ix^—Na4—Na1^xii^	114.701 (16)
O7—Na1—Na4^iii^	41.342 (13)	O8^ix^—Na4—Na1^xii^	34.668 (13)
O5^iii^—Na1—Na4^iii^	39.891 (12)	O2^x^—Na4—Na1^xii^	75.547 (14)
O5^ii^—Na1—Na4^iii^	104.346 (15)	O3^x^—Na4—Na1^xii^	103.732 (14)
S4^iii^—Na1—Na4^iii^	87.283 (9)	S1^x^—Na4—Na1^xii^	90.121 (9)
S3^ii^—Na1—Na4^iii^	97.020 (10)	S4^xi^—Na4—Na1^xii^	46.906 (7)
S3^iii^—Na1—Na4^iii^	59.736 (7)	S2^vii^—Na4—Na1^xii^	166.892 (10)
S4^iv^—Na1—Na4^iii^	168.666 (11)	Na1^ix^—Na4—Na1^xii^	99.823 (10)
O8^i^—Na1—Na4^v^	35.554 (14)	O5—Na4—Na3^x^	153.064 (17)
O6^ii^—Na1—Na4^v^	152.272 (16)	O7^ix^—Na4—Na3^x^	71.212 (15)
O7—Na1—Na4^v^	80.915 (15)	O8^ix^—Na4—Na3^x^	73.120 (14)
O5^iii^—Na1—Na4^v^	104.647 (15)	O2^x^—Na4—Na3^x^	34.492 (11)
O5^ii^—Na1—Na4^v^	142.833 (15)	O3^x^—Na4—Na3^x^	81.785 (13)
S4^iii^—Na1—Na4^v^	53.522 (7)	S1^x^—Na4—Na3^x^	56.206 (7)
S3^ii^—Na1—Na4^v^	163.151 (11)	S4^xi^—Na4—Na3^x^	128.605 (10)
S3^iii^—Na1—Na4^v^	85.734 (8)	S2^vii^—Na4—Na3^x^	83.368 (8)
S4^iv^—Na1—Na4^v^	88.121 (8)	Na1^ix^—Na4—Na3^x^	110.847 (10)
Na4^iii^—Na1—Na4^v^	99.823 (10)	Na1^xii^—Na4—Na3^x^	90.440 (9)
O8^i^—Na1—Na1^vi^	162.27 (2)	O5—Na4—Na3^xi^	98.583 (15)
O6^ii^—Na1—Na1^vi^	74.472 (14)	O7^ix^—Na4—Na3^xi^	166.615 (18)
O7—Na1—Na1^vi^	111.295 (17)	O8^ix^—Na4—Na3^xi^	112.225 (16)
O5^iii^—Na1—Na1^vi^	40.314 (13)	O2^x^—Na4—Na3^xi^	74.432 (12)
O5^ii^—Na1—Na1^vi^	37.803 (12)	O3^x^—Na4—Na3^xi^	36.423 (12)
S4^iii^—Na1—Na1^vi^	79.880 (10)	S1^x^—Na4—Na3^xi^	52.850 (6)
S3^ii^—Na1—Na1^vi^	52.954 (7)	S4^xi^—Na4—Na3^xi^	46.673 (6)
S3^iii^—Na1—Na1^vi^	49.554 (7)	S2^vii^—Na4—Na3^xi^	92.385 (8)
S4^iv^—Na1—Na1^vi^	97.138 (11)	Na1^ix^—Na4—Na3^xi^	141.291 (10)
Na4^iii^—Na1—Na1^vi^	71.532 (10)	Na1^xii^—Na4—Na3^xi^	78.487 (8)
Na4^v^—Na1—Na1^vi^	133.254 (12)	Na3^x^—Na4—Na3^xi^	107.842 (8)
O1—Na2—O6^ii^	82.24 (2)	O1—S1—O3	111.15 (3)
O1—Na2—O9^ii^	121.57 (2)	O1—S1—O2	110.51 (3)
O6^ii^—Na2—O9^ii^	119.40 (2)	O3—S1—O2	109.00 (3)
O1—Na2—O9^i^	150.70 (2)	O1—S1—S2	108.98 (2)
O6^ii^—Na2—O9^i^	98.37 (2)	O3—S1—S2	107.713 (19)
O9^ii^—Na2—O9^i^	83.83 (2)	O2—S1—S2	109.43 (2)
O1—Na2—O10	82.508 (19)	O1—S1—Na4^viii^	126.09 (2)
O6^ii^—Na2—O10	155.73 (2)	O3—S1—Na4^viii^	55.78 (2)
O9^ii^—Na2—O10	84.74 (2)	O2—S1—Na4^viii^	53.25 (2)
O9^i^—Na2—O10	85.998 (19)	S2—S1—Na4^viii^	124.926 (9)
O1—Na2—Na2^iv^	159.85 (2)	O1—S1—Na3^xiii^	133.51 (2)
O6^ii^—Na2—Na2^iv^	115.254 (19)	O3—S1—Na3^xiii^	46.299 (19)
O9^ii^—Na2—Na2^iv^	42.140 (14)	O2—S1—Na3^xiii^	115.357 (19)
O9^i^—Na2—Na2^iv^	41.695 (14)	S2—S1—Na3^xiii^	62.288 (7)
O10—Na2—Na2^iv^	83.775 (16)	Na4^viii^—S1—Na3^xiii^	78.992 (8)
O1—Na2—Na3	58.132 (15)	S1—S2—Na3^xiii^	80.713 (8)
O6^ii^—Na2—Na3	139.920 (17)	S1—S2—Na4^xiii^	123.039 (9)
O9^ii^—Na2—Na3	81.357 (15)	Na3^xiii^—S2—Na4^xiii^	94.364 (8)
O9^i^—Na2—Na3	118.828 (17)	S1—O1—Na2	146.31 (3)
O10—Na2—Na3	33.841 (13)	S1—O2—Na3	122.37 (3)
Na2^iv^—Na2—Na3	102.995 (12)	S1—O2—Na4^viii^	98.24 (2)
O1—Na2—Na1	90.813 (17)	Na3—O2—Na4^viii^	109.004 (19)
O6^ii^—Na2—Na1	33.978 (14)	S1—O3—Na3^xiii^	108.43 (2)
O9^ii^—Na2—Na1	138.631 (17)	S1—O3—Na4^viii^	95.58 (2)
O9^i^—Na2—Na1	75.239 (16)	Na3^xiii^—O3—Na4^viii^	106.34 (2)
O10—Na2—Na1	127.834 (17)	O4—S3—O5	111.71 (3)
Na2^iv^—Na2—Na1	109.277 (12)	O4—S3—O6	110.90 (3)
Na3—Na2—Na1	140.011 (10)	O5—S3—O6	108.66 (3)
O1—Na2—H8	78.8 (3)	O4—S3—S4	107.82 (2)
O6^ii^—Na2—H8	139.2 (3)	O5—S3—S4	108.64 (2)
O9^ii^—Na2—H8	101.3 (3)	O6—S3—S4	109.05 (2)
O9^i^—Na2—H8	82.1 (3)	O4—S3—Na1^ii^	129.57 (2)
O10—Na2—H8	17.5 (3)	O5—S3—Na1^ii^	57.81 (2)
Na2^iv^—Na2—H8	92.2 (3)	O6—S3—Na1^ii^	50.86 (2)
Na3—Na2—H8	44.3 (3)	S4—S3—Na1^ii^	122.440 (9)
Na1—Na2—H8	110.5 (3)	O4—S3—Na3	44.20 (2)
O10—Na3—O2	92.728 (19)	O5—S3—Na3	115.89 (2)
O10—Na3—O4	113.74 (2)	O6—S3—Na3	134.61 (2)
O2—Na3—O4	100.217 (19)	S4—S3—Na3	64.862 (7)
O10—Na3—O3^vii^	91.184 (18)	Na1^ii^—S3—Na3	170.840 (9)
O2—Na3—O3^vii^	112.831 (19)	O4—S3—Na1^ix^	135.24 (3)
O4—Na3—O3^vii^	137.58 (2)	O5—S3—Na1^ix^	47.07 (2)
O10—Na3—S2^vii^	152.055 (17)	O6—S3—Na1^ix^	113.42 (2)
O2—Na3—S2^vii^	91.337 (14)	S4—S3—Na1^ix^	62.814 (7)
O4—Na3—S2^vii^	92.654 (19)	Na1^ii^—S3—Na1^ix^	77.492 (9)
O3^vii^—Na3—S2^vii^	61.898 (12)	Na3—S3—Na1^ix^	103.191 (8)
O10—Na3—S4	83.176 (15)	S3—S4—Na1^ix^	79.685 (8)
O2—Na3—S4	157.854 (16)	S3—S4—Na3	77.566 (8)
O4—Na3—S4	62.368 (14)	Na1^ix^—S4—Na3	117.727 (9)
O3^vii^—Na3—S4	89.069 (14)	S3—S4—Na4^i^	138.838 (9)
S2^vii^—Na3—S4	102.479 (9)	Na1^ix^—S4—Na4^i^	79.571 (9)
O10—Na3—S3	106.368 (16)	Na3—S4—Na4^i^	81.298 (8)
O2—Na3—S3	125.470 (15)	S3—S4—Na1^iv^	127.876 (9)
O4—Na3—S3	25.345 (12)	Na1^ix^—S4—Na1^iv^	88.951 (9)
O3^vii^—Na3—S3	117.147 (15)	Na3—S4—Na1^iv^	147.809 (9)
S2^vii^—Na3—S3	93.535 (8)	Na4^i^—S4—Na1^iv^	86.748 (8)
S4—Na3—S3	37.573 (5)	S3—O4—Na3	110.46 (3)
O10—Na3—S1^vii^	116.459 (16)	S3—O5—Na4	126.64 (3)
O2—Na3—S1^vii^	109.280 (14)	S3—O5—Na1^ix^	107.13 (2)
O4—Na3—S1^vii^	119.167 (19)	Na4—O5—Na1^ix^	97.307 (19)
O3^vii^—Na3—S1^vii^	25.276 (10)	S3—O5—Na1^ii^	93.72 (2)
S2^vii^—Na3—S1^vii^	36.999 (5)	Na4—O5—Na1^ii^	126.97 (2)
S4—Na3—S1^vii^	91.884 (8)	Na1^ix^—O5—Na1^ii^	101.88 (2)
S3—Na3—S1^vii^	106.583 (9)	S3—O6—Na2^ii^	126.21 (3)
O10—Na3—Na2	35.370 (14)	S3—O6—Na1^ii^	101.14 (3)
O2—Na3—Na2	80.167 (14)	Na2^ii^—O6—Na1^ii^	113.05 (2)
O4—Na3—Na2	83.528 (19)	Na4^iii^—O7—Na1	96.27 (2)
O3^vii^—Na3—Na2	126.537 (14)	Na4^iii^—O7—H1	114.1 (9)
S2^vii^—Na3—Na2	169.873 (10)	Na1—O7—H1	106.9 (9)
S4—Na3—Na2	84.098 (8)	Na4^iii^—O7—H2	113.1 (9)
S3—Na3—Na2	87.028 (8)	Na1—O7—H2	121.0 (9)
S1^vii^—Na3—Na2	151.796 (10)	H1—O7—H2	105.6 (12)
O10—Na3—Na4^viii^	76.972 (15)	Na1^xi^—O8—Na4^iii^	109.78 (2)
O2—Na3—Na4^viii^	36.505 (13)	Na1^xi^—O8—H3	114.0 (9)
O4—Na3—Na4^viii^	136.702 (15)	Na4^iii^—O8—H3	109.3 (9)
O3^vii^—Na3—Na4^viii^	80.289 (14)	Na1^xi^—O8—H4	100.8 (13)
S2^vii^—Na3—Na4^viii^	90.294 (9)	Na4^iii^—O8—H4	114.9 (13)
S4—Na3—Na4^viii^	157.179 (10)	H3—O8—H4	107.9 (15)
S3—Na3—Na4^viii^	161.760 (10)	Na2^ii^—O9—Na2^xi^	96.16 (2)
S1^vii^—Na3—Na4^viii^	87.044 (8)	Na2^ii^—O9—H5	128.6 (13)
Na2—Na3—Na4^viii^	86.141 (8)	Na2^xi^—O9—H5	112.6 (12)
O10—Na3—Na4^i^	83.574 (15)	Na2^ii^—O9—H6	108.2 (10)
O2—Na3—Na4^i^	149.322 (16)	Na2^xi^—O9—H6	111.5 (9)
O4—Na3—Na4^i^	109.170 (15)	H5—O9—H6	99.8 (14)
O3^vii^—Na3—Na4^i^	37.235 (13)	Na3—O10—Na2	110.79 (2)
S2^vii^—Na3—Na4^i^	78.954 (8)	Na3—O10—H7	111.9 (9)
S4—Na3—Na4^i^	52.030 (6)	Na2—O10—H7	118.0 (9)
S3—Na3—Na4^i^	84.462 (8)	Na3—O10—H8	114.5 (10)
S1^vii^—Na3—Na4^i^	48.157 (6)	Na2—O10—H8	92.0 (10)
Na2—Na3—Na4^i^	111.153 (9)	H7—O10—H8	108.4 (13)
Na4^viii^—Na3—Na4^i^	113.778 (9)		

Symmetry codes: (i) *x*+1, *y*, *z*; (ii) −*x*+1, −*y*, −*z*+1; (iii) *x*, *y*, *z*−1; (iv) −*x*+2, −*y*, −*z*+1; (v) *x*+1, *y*, *z*−1; (vi) −*x*+1, −*y*, −*z*; (vii) *x*+1/2, −*y*+1/2, *z*+1/2; (viii) *x*+1/2, −*y*+1/2, *z*−1/2; (ix) *x*, *y*, *z*+1; (x) *x*−1/2, −*y*+1/2, *z*+1/2; (xi) *x*−1, *y*, *z*; (xii) *x*−1, *y*, *z*+1; (xiii) *x*−1/2, −*y*+1/2, *z*−1/2.

### (Na2S2O3H2O2_200K) Hydrogen-bond geometry (Å, º)

**Table:**

*D*—H···*A*	*D*—H	H···*A*	*D*···*A*	*D*—H···*A*
O7—H1···O2^viii^	0.847 (13)	2.105 (13)	2.9400 (7)	168.4 (12)
O7—H2···O3	0.795 (13)	2.183 (13)	2.9595 (7)	165.6 (13)
O8—H3···O6^ii^	0.804 (13)	2.303 (14)	3.0991 (7)	170.8 (12)
O8—H4···S2	0.721 (17)	2.600 (17)	3.3188 (5)	175.7 (17)
O9—H5···O1	0.835 (17)	1.994 (18)	2.8237 (7)	172.4 (18)
O9—H6···S4^ii^	0.808 (14)	2.499 (14)	3.3069 (5)	178.4 (12)
O10—H7···O4^i^	0.835 (14)	1.930 (14)	2.7602 (7)	172.5 (13)
O10—H8···S2^i^	0.772 (14)	2.469 (14)	3.2261 (5)	166.9 (13)

Symmetry codes: (i) *x*+1, *y*, *z*; (ii) −*x*+1, −*y*, −*z*+1; (viii) *x*+1/2, −*y*+1/2, *z*−1/2.

### (Na2S2O3H2O5_100K) Crystal data

**Table:**

O_3_S_2_·5(H_2_O)·2(Na)	*F*(000) = 512
*M**_r_* = 248.18	*D*_x_ = 1.777 Mg m^−^^3^
Monoclinic, *P*2_1_/*c*	Mo *K*α radiation, λ = 0.71073 Å
*a* = 5.9187 (1) Å	Cell parameters from 69507 reflections
*b* = 21.5173 (4) Å	θ = 3.0–42.4°
*c* = 7.4979 (1) Å	µ = 0.67 mm^−^^1^
β = 103.722 (1)°	*T* = 100 K
*V* = 927.64 (3) Å^3^	Block, colourless
*Z* = 4	0.6 × 0.3 × 0.15 mm

### (Na2S2O3H2O5_100K) Data collection

**Table:**

Stoe StadiVari diffractometer	6486 independent reflections
Radiation source: Genix 3D HF Mo	5525 reflections with *I* > 2σ(*I*)
Graded multilayer mirror monochromator	*R*_int_ = 0.026
Detector resolution: 5.81 pixels mm^-1^	θ_max_ = 42.3°, θ_min_ = 3.0°
ω scans	*h* = −11→11
Absorption correction: empirical (using intensity measurements) (X-AREA; Stoe & Cie, 2015)	*k* = −40→40
*T*_min_ = 0.868, *T*_max_ = 1.000	*l* = −14→9
64240 measured reflections	

### (Na2S2O3H2O5_100K) Refinement

**Table:**

Refinement on *F*^2^	Primary atom site location: structure-invariant direct methods
Least-squares matrix: full	Secondary atom site location: difference Fourier map
*R*[*F*^2^ > 2σ(*F*^2^)] = 0.020	Hydrogen site location: difference Fourier map
*wR*(*F*^2^) = 0.047	All H-atom parameters refined
*S* = 1.06	*w* = 1/[σ^2^(*F*_o_^2^) + (0.0269*P*)^2^ + 0.0151*P*] where *P* = (*F*_o_^2^ + 2*F*_c_^2^)/3
6486 reflections	(Δ/σ)_max_ = 0.001
149 parameters	Δρ_max_ = 0.54 e Å^−^^3^
0 restraints	Δρ_min_ = −0.27 e Å^−^^3^

### (Na2S2O3H2O5_100K) Special details

**Table:**

Geometry. All esds (except the esd in the dihedral angle between two l.s. planes) are estimated using the full covariance matrix. The cell esds are taken into account individually in the estimation of esds in distances, angles and torsion angles; correlations between esds in cell parameters are only used when they are defined by crystal symmetry. An approximate (isotropic) treatment of cell esds is used for estimating esds involving l.s. planes.

### (Na2S2O3H2O5_100K) Fractional atomic coordinates and isotropic or equivalent isotropic displacement parameters (Å^2^)

**Table:**

	*x*	*y*	*z*	*U*_iso_*/*U*_eq_	
Na1	0.72487 (4)	0.34133 (2)	0.07542 (3)	0.01006 (3)	
Na2	0.25504 (4)	0.40853 (2)	0.21568 (3)	0.01143 (4)	
S1	0.14781 (2)	0.14166 (2)	0.27653 (2)	0.00683 (2)	
S2	0.10308 (2)	0.06742 (2)	0.10544 (2)	0.00895 (2)	
O1	0.33884 (6)	0.12823 (2)	0.43602 (5)	0.01248 (6)	
O2	−0.07043 (6)	0.15368 (2)	0.33255 (5)	0.01157 (5)	
O3	0.20340 (6)	0.19572 (2)	0.17106 (5)	0.01064 (5)	
O4	0.62384 (6)	0.23471 (2)	0.09598 (6)	0.01306 (6)	
H1	0.505 (2)	0.2244 (6)	0.1222 (17)	0.029 (3)*	
H2	0.7229 (19)	0.2157 (6)	0.1692 (16)	0.021 (2)*	
O5	0.09097 (6)	0.31382 (2)	0.27759 (5)	0.01099 (5)	
H3	0.132 (2)	0.2794 (6)	0.2504 (17)	0.029 (3)*	
H4	0.104 (2)	0.3116 (6)	0.3873 (18)	0.026 (3)*	
O6	0.61660 (6)	0.36783 (2)	0.35694 (5)	0.01218 (6)	
H5	0.620 (2)	0.3354 (7)	0.4218 (18)	0.032 (3)*	
H6	0.736 (2)	0.3872 (6)	0.4162 (17)	0.029 (3)*	
O7	0.86492 (7)	0.44750 (2)	0.10769 (5)	0.01232 (5)	
H7	0.849 (2)	0.4768 (6)	0.1746 (18)	0.028 (3)*	
H8	0.806 (2)	0.4592 (6)	0.0042 (17)	0.026 (3)*	
O8	0.64754 (6)	0.01526 (2)	0.24596 (6)	0.01309 (6)	
H9	0.733 (2)	0.0319 (6)	0.1941 (18)	0.032 (3)*	
H10	0.524 (2)	0.0297 (6)	0.2069 (18)	0.033 (3)*	

### (Na2S2O3H2O5_100K) Atomic displacement parameters (Å^2^)

**Table:**

	*U*^11^	*U*^22^	*U*^33^	*U*^12^	*U*^13^	*U*^23^
Na1	0.00909 (7)	0.01107 (8)	0.00987 (8)	−0.00034 (6)	0.00196 (6)	−0.00033 (6)
Na2	0.00991 (8)	0.01013 (8)	0.01364 (9)	0.00026 (6)	0.00160 (6)	0.00022 (6)
S1	0.00648 (3)	0.00759 (4)	0.00631 (4)	−0.00005 (3)	0.00127 (3)	0.00026 (3)
S2	0.01010 (4)	0.00757 (4)	0.00881 (4)	−0.00018 (3)	0.00151 (3)	−0.00055 (3)
O1	0.00991 (12)	0.01622 (14)	0.00904 (13)	0.00008 (10)	−0.00225 (9)	0.00048 (10)
O2	0.00971 (12)	0.01476 (14)	0.01171 (13)	0.00181 (10)	0.00544 (10)	0.00062 (10)
O3	0.01410 (13)	0.00786 (12)	0.01111 (13)	−0.00165 (10)	0.00524 (10)	0.00061 (9)
O4	0.01005 (12)	0.01317 (14)	0.01548 (15)	−0.00037 (10)	0.00204 (10)	0.00128 (11)
O5	0.01314 (13)	0.00956 (12)	0.00996 (13)	0.00146 (10)	0.00211 (10)	−0.00052 (9)
O6	0.00987 (12)	0.01442 (14)	0.01149 (14)	−0.00074 (10)	0.00105 (10)	0.00104 (10)
O7	0.01419 (13)	0.01036 (13)	0.01148 (14)	0.00069 (10)	0.00118 (10)	−0.00052 (10)
O8	0.01060 (13)	0.01258 (14)	0.01597 (15)	0.00096 (10)	0.00292 (11)	0.00182 (11)

### (Na2S2O3H2O5_100K) Geometric parameters (Å, º)

**Table:**

Na1—O1^i^	2.3682 (4)	S1—S2	2.0266 (1)
Na1—O4	2.3850 (4)	S1—Na2^vi^	3.3793 (2)
Na1—O5^ii^	2.4052 (4)	S2—Na2^i^	3.2961 (3)
Na1—O6	2.4152 (4)	O1—Na1^vi^	2.3683 (4)
Na1—O2^iii^	2.4170 (4)	O1—Na2^vi^	2.4004 (4)
Na1—O7	2.4227 (4)	O2—Na1^vii^	2.4170 (4)
Na1—Na2^ii^	3.3879 (3)	O4—H1	0.808 (12)
Na1—Na2	3.5095 (3)	O4—H2	0.812 (12)
Na1—H8	2.658 (12)	O5—Na1^v^	2.4052 (4)
Na2—O6	2.3223 (4)	O5—H3	0.820 (13)
Na2—O5	2.3506 (4)	O5—H4	0.809 (13)
Na2—O8^iv^	2.3686 (4)	O6—H5	0.847 (14)
Na2—O1^i^	2.4004 (4)	O6—H6	0.850 (13)
Na2—O7^v^	2.4090 (4)	O7—Na2^ii^	2.4090 (4)
Na2—S2^vi^	3.2960 (3)	O7—H7	0.824 (13)
Na2—S1^i^	3.3793 (2)	O7—H8	0.812 (13)
Na2—Na1^v^	3.3879 (3)	O8—Na2^viii^	2.3687 (4)
S1—O1	1.4665 (4)	O8—H9	0.792 (13)
S1—O2	1.4728 (3)	O8—H10	0.785 (14)
S1—O3	1.4867 (4)		
			
O1^i^—Na1—O4	93.674 (15)	S2^vi^—Na2—S1^i^	152.425 (7)
O1^i^—Na1—O5^ii^	167.632 (16)	O6—Na2—Na1^v^	131.763 (13)
O4—Na1—O5^ii^	85.687 (14)	O5—Na2—Na1^v^	45.224 (10)
O1^i^—Na1—O6	83.813 (14)	O8^iv^—Na2—Na1^v^	129.225 (13)
O4—Na1—O6	92.752 (15)	O1^i^—Na2—Na1^v^	87.492 (11)
O5^ii^—Na1—O6	83.881 (14)	O7^v^—Na2—Na1^v^	45.643 (10)
O1^i^—Na1—O2^iii^	104.997 (15)	S2^vi^—Na2—Na1^v^	85.113 (6)
O4—Na1—O2^iii^	105.640 (15)	S1^i^—Na2—Na1^v^	67.361 (6)
O5^ii^—Na1—O2^iii^	87.024 (14)	O6—Na2—Na1	43.228 (11)
O6—Na1—O2^iii^	158.798 (16)	O5—Na2—Na1	95.497 (12)
O1^i^—Na1—O7	93.139 (15)	O8^iv^—Na2—Na1	104.388 (12)
O4—Na1—O7	170.292 (17)	O1^i^—Na2—Na1	42.257 (10)
O5^ii^—Na1—O7	86.177 (14)	O7^v^—Na2—Na1	143.813 (13)
O6—Na1—O7	81.115 (14)	S2^vi^—Na2—Na1	137.109 (8)
O2^iii^—Na1—O7	79.197 (14)	S1^i^—Na2—Na1	63.600 (6)
O1^i^—Na1—Na2^ii^	138.122 (13)	Na1^v^—Na2—Na1	118.197 (9)
O4—Na1—Na2^ii^	128.202 (12)	O1—S1—O2	111.14 (2)
O5^ii^—Na1—Na2^ii^	43.924 (10)	O1—S1—O3	111.28 (2)
O6—Na1—Na2^ii^	92.647 (11)	O2—S1—O3	109.52 (2)
O2^iii^—Na1—Na2^ii^	67.791 (11)	O1—S1—S2	108.693 (17)
O7—Na1—Na2^ii^	45.314 (10)	O2—S1—S2	109.098 (16)
O1^i^—Na1—Na2	42.968 (10)	O3—S1—S2	106.994 (15)
O4—Na1—Na2	98.530 (12)	O1—S1—Na2^vi^	38.016 (16)
O5^ii^—Na1—Na2	124.885 (12)	O2—S1—Na2^vi^	75.695 (16)
O6—Na1—Na2	41.191 (10)	O3—S1—Na2^vi^	138.961 (15)
O2^iii^—Na1—Na2	141.432 (13)	S2—S1—Na2^vi^	109.327 (6)
O7—Na1—Na2	81.849 (11)	S1—S2—Na2^i^	114.539 (6)
Na2^ii^—Na1—Na2	118.197 (9)	S1—O1—Na1^vi^	141.43 (2)
O1^i^—Na1—H8	81.3 (3)	S1—O1—Na2^vi^	119.88 (2)
O4—Na1—H8	172.1 (3)	Na1^vi^—O1—Na2^vi^	94.775 (14)
O5^ii^—Na1—H8	100.6 (3)	S1—O2—Na1^vii^	148.53 (2)
O6—Na1—H8	92.8 (3)	Na1—O4—H1	121.7 (9)
O2^iii^—Na1—H8	70.1 (3)	Na1—O4—H2	112.1 (8)
O7—Na1—H8	17.6 (3)	H1—O4—H2	103.3 (12)
Na2^ii^—Na1—H8	57.2 (3)	Na2—O5—Na1^v^	90.850 (14)
Na2—Na1—H8	82.0 (3)	Na2—O5—H3	124.9 (9)
O6—Na2—O5	87.830 (15)	Na1^v^—O5—H3	110.0 (9)
O6—Na2—O8^iv^	97.981 (16)	Na2—O5—H4	108.0 (9)
O5—Na2—O8^iv^	156.443 (17)	Na1^v^—O5—H4	120.6 (9)
O6—Na2—O1^i^	85.131 (15)	H3—O5—H4	103.7 (12)
O5—Na2—O1^i^	93.865 (15)	Na2—O6—Na1	95.581 (15)
O8^iv^—Na2—O1^i^	109.318 (16)	Na2—O6—H5	117.9 (9)
O6—Na2—O7^v^	172.157 (17)	Na1—O6—H5	109.3 (9)
O5—Na2—O7^v^	87.719 (15)	Na2—O6—H6	128.0 (9)
O8^iv^—Na2—O7^v^	83.664 (14)	Na1—O6—H6	102.3 (8)
O1^i^—Na2—O7^v^	101.611 (15)	H5—O6—H6	101.4 (12)
O6—Na2—S2^vi^	94.048 (12)	Na2^ii^—O7—Na1	89.042 (14)
O5—Na2—S2^vi^	75.443 (11)	Na2^ii^—O7—H7	107.4 (9)
O8^iv^—Na2—S2^vi^	81.366 (12)	Na1—O7—H7	134.1 (9)
O1^i^—Na2—S2^vi^	169.304 (13)	Na2^ii^—O7—H8	126.3 (8)
O7^v^—Na2—S2^vi^	78.579 (11)	Na1—O7—H8	97.8 (9)
O6—Na2—S1^i^	105.285 (12)	H7—O7—H8	105.0 (12)
O5—Na2—S1^i^	85.665 (11)	Na2^viii^—O8—H9	109.5 (10)
O8^iv^—Na2—S1^i^	114.344 (13)	Na2^viii^—O8—H10	127.3 (10)
O1^i^—Na2—S1^i^	22.102 (9)	H9—O8—H10	106.5 (13)
O7^v^—Na2—S1^i^	80.806 (11)		

Symmetry codes: (i) *x*, −*y*+1/2, *z*−1/2; (ii) *x*+1, *y*, *z*; (iii) *x*+1, −*y*+1/2, *z*−1/2; (iv) −*x*+1, *y*+1/2, −*z*+1/2; (v) *x*−1, *y*, *z*; (vi) *x*, −*y*+1/2, *z*+1/2; (vii) *x*−1, −*y*+1/2, *z*+1/2; (viii) −*x*+1, *y*−1/2, −*z*+1/2.

### (Na2S2O3H2O5_100K) Hydrogen-bond geometry (Å, º)

**Table:**

*D*—H···*A*	*D*—H	H···*A*	*D*···*A*	*D*—H···*A*
O4—H1···O3	0.808 (12)	2.001 (12)	2.8067 (5)	176.1 (13)
O4—H2···O2^ii^	0.812 (12)	2.017 (12)	2.8175 (5)	168.6 (12)
O5—H3···O3	0.820 (13)	1.973 (13)	2.7912 (5)	174.9 (12)
O5—H4···O3^vi^	0.809 (13)	2.074 (13)	2.8736 (5)	169.2 (12)
O6—H5···O4^vi^	0.847 (14)	1.993 (14)	2.8365 (6)	173.8 (13)
O6—H6···S2^ix^	0.850 (13)	2.495 (13)	3.3404 (4)	173.6 (12)
O7—H7···S2^iv^	0.824 (13)	2.527 (13)	3.3356 (4)	167.5 (11)
O7—H8···O8^i^	0.812 (13)	2.017 (13)	2.8280 (6)	177.2 (12)
O8—H9···S2^ii^	0.792 (13)	2.554 (13)	3.3147 (4)	161.6 (12)
O8—H10···S2	0.785 (14)	2.558 (14)	3.3381 (4)	173.0 (13)

Symmetry codes: (i) *x*, −*y*+1/2, *z*−1/2; (ii) *x*+1, *y*, *z*; (iv) −*x*+1, *y*+1/2, −*z*+1/2; (vi) *x*, −*y*+1/2, *z*+1/2; (ix) *x*+1, −*y*+1/2, *z*+1/2.

### (Na2S2O3H2O5_200K) Crystal data

**Table:**

O_3_S_2_·5(H_2_O)·2(Na)	*F*(000) = 512
*M**_r_* = 248.18	*D*_x_ = 1.769 Mg m^−^^3^
Monoclinic, *P*2_1_/*c*	Mo *K*α radiation, λ = 0.71073 Å
*a* = 5.9357 (1) Å	Cell parameters from 72911 reflections
*b* = 21.5424 (7) Å	θ = 3.4–38.4°
*c* = 7.5026 (2) Å	µ = 0.67 mm^−^^1^
β = 103.722 (2)°	*T* = 200 K
*V* = 931.97 (4) Å^3^	Block, colourless
*Z* = 4	0.6 × 0.3 × 0.15 mm

### (Na2S2O3H2O5_200K) Data collection

**Table:**

Stoe StadiVari diffractometer	5006 independent reflections
Radiation source: Genix 3D HF Mo	4212 reflections with *I* > 2σ(*I*)
Graded multilayer mirror monochromator	*R*_int_ = 0.023
Detector resolution: 5.81 pixels mm^-1^	θ_max_ = 38.1°, θ_min_ = 3.4°
ω scans	*h* = −10→10
Absorption correction: empirical (using intensity measurements) (X-AREA; Stoe & Cie, 2015)	*k* = −37→37
*T*_min_ = 0.869, *T*_max_ = 1.000	*l* = −12→12
56700 measured reflections	

### (Na2S2O3H2O5_200K) Refinement

**Table:**

Refinement on *F*^2^	Primary atom site location: structure-invariant direct methods
Least-squares matrix: full	Secondary atom site location: difference Fourier map
*R*[*F*^2^ > 2σ(*F*^2^)] = 0.019	Hydrogen site location: difference Fourier map
*wR*(*F*^2^) = 0.052	All H-atom parameters refined
*S* = 1.08	*w* = 1/[σ^2^(*F*_o_^2^) + (0.0284*P*)^2^ + 0.0519*P*] where *P* = (*F*_o_^2^ + 2*F*_c_^2^)/3
5006 reflections	(Δ/σ)_max_ = 0.001
149 parameters	Δρ_max_ = 0.42 e Å^−^^3^
0 restraints	Δρ_min_ = −0.28 e Å^−^^3^

### (Na2S2O3H2O5_200K) Special details

**Table:**

Geometry. All esds (except the esd in the dihedral angle between two l.s. planes) are estimated using the full covariance matrix. The cell esds are taken into account individually in the estimation of esds in distances, angles and torsion angles; correlations between esds in cell parameters are only used when they are defined by crystal symmetry. An approximate (isotropic) treatment of cell esds is used for estimating esds involving l.s. planes.

### (Na2S2O3H2O5_200K) Fractional atomic coordinates and isotropic or equivalent isotropic displacement parameters (Å^2^)

**Table:**

	*x*	*y*	*z*	*U*_iso_*/*U*_eq_	
Na1	0.72432 (4)	0.34120 (2)	0.07497 (3)	0.01737 (5)	
Na2	0.25469 (5)	0.40844 (2)	0.21534 (4)	0.02015 (5)	
S1	0.14796 (2)	0.14165 (2)	0.27688 (2)	0.01199 (3)	
S2	0.10261 (3)	0.06772 (2)	0.10611 (2)	0.01584 (3)	
O1	0.33851 (8)	0.12808 (3)	0.43550 (6)	0.02186 (9)	
O2	−0.06889 (8)	0.15357 (2)	0.33334 (7)	0.02022 (9)	
O3	0.20282 (8)	0.19559 (2)	0.17211 (6)	0.01850 (8)	
O4	0.62336 (9)	0.23466 (2)	0.09666 (7)	0.02250 (9)	
H1	0.510 (3)	0.2256 (6)	0.1234 (19)	0.050 (4)*	
H2	0.722 (2)	0.2159 (5)	0.1725 (16)	0.029 (3)*	
O5	0.09082 (9)	0.31379 (2)	0.27781 (7)	0.01859 (8)	
H3	0.135 (2)	0.2789 (6)	0.2509 (17)	0.041 (3)*	
H4	0.100 (2)	0.3107 (6)	0.3837 (18)	0.037 (3)*	
O6	0.61589 (8)	0.36779 (3)	0.35662 (7)	0.02082 (9)	
H5	0.616 (2)	0.3358 (6)	0.4249 (18)	0.042 (3)*	
H6	0.734 (2)	0.3882 (6)	0.4170 (17)	0.046 (3)*	
O7	0.86448 (9)	0.44752 (2)	0.10841 (7)	0.02104 (9)	
H7	0.851 (2)	0.4775 (6)	0.1719 (18)	0.042 (3)*	
H8	0.801 (2)	0.4599 (6)	−0.0005 (19)	0.042 (3)*	
O8	0.64707 (9)	0.01512 (2)	0.24637 (8)	0.02268 (9)	
H9	0.734 (3)	0.0327 (7)	0.194 (2)	0.053 (4)*	
H10	0.528 (3)	0.0308 (6)	0.2099 (19)	0.049 (4)*	

### (Na2S2O3H2O5_200K) Atomic displacement parameters (Å^2^)

**Table:**

	*U*^11^	*U*^22^	*U*^33^	*U*^12^	*U*^13^	*U*^23^
Na1	0.01558 (11)	0.01926 (12)	0.01702 (11)	−0.00067 (9)	0.00338 (9)	−0.00062 (8)
Na2	0.01729 (12)	0.01729 (12)	0.02491 (13)	0.00058 (9)	0.00308 (9)	0.00077 (9)
S1	0.01134 (5)	0.01321 (6)	0.01121 (5)	−0.00010 (4)	0.00228 (4)	0.00042 (4)
S2	0.01840 (6)	0.01296 (6)	0.01544 (6)	−0.00045 (4)	0.00258 (5)	−0.00103 (4)
O1	0.01746 (19)	0.0281 (2)	0.01595 (18)	0.00009 (17)	−0.00416 (15)	0.00017 (16)
O2	0.01731 (19)	0.0257 (2)	0.02027 (19)	0.00338 (16)	0.00971 (16)	0.00130 (16)
O3	0.0247 (2)	0.01328 (18)	0.01982 (19)	−0.00270 (15)	0.00978 (16)	0.00071 (14)
O4	0.0169 (2)	0.0227 (2)	0.0272 (2)	−0.00065 (17)	0.00376 (18)	0.00230 (18)
O5	0.0227 (2)	0.01597 (19)	0.01668 (18)	0.00263 (16)	0.00376 (15)	−0.00085 (14)
O6	0.01690 (19)	0.0251 (2)	0.0192 (2)	−0.00092 (17)	0.00170 (16)	0.00212 (16)
O7	0.0241 (2)	0.0170 (2)	0.0204 (2)	0.00113 (17)	0.00206 (17)	−0.00118 (16)
O8	0.0185 (2)	0.0208 (2)	0.0287 (2)	0.00186 (17)	0.00558 (18)	0.00340 (17)

### (Na2S2O3H2O5_200K) Geometric parameters (Å, º)

**Table:**

Na1—O1^i^	2.3750 (5)	S1—S2	2.0216 (2)
Na1—O4	2.3872 (6)	S1—Na2^vi^	3.3761 (3)
Na1—O5^ii^	2.4138 (6)	S2—Na2^i^	3.3060 (3)
Na1—O6	2.4193 (6)	O1—Na1^vi^	2.3750 (5)
Na1—O2^iii^	2.4210 (5)	O1—Na2^vi^	2.4020 (6)
Na1—O7	2.4294 (6)	O2—Na1^vii^	2.4211 (5)
Na1—Na2^ii^	3.3978 (4)	O4—H1	0.774 (15)
Na1—Na2	3.5172 (4)	O4—H2	0.820 (12)
Na2—O6	2.3256 (6)	O5—Na1^v^	2.4138 (6)
Na2—O5	2.3536 (6)	O5—H3	0.836 (13)
Na2—O8^iv^	2.3717 (6)	O5—H4	0.786 (13)
Na2—O1^i^	2.4021 (6)	O6—H5	0.859 (14)
Na2—O7^v^	2.4157 (6)	O6—H6	0.860 (14)
Na2—S2^vi^	3.3060 (3)	O7—Na2^ii^	2.4157 (6)
Na2—S1^i^	3.3761 (3)	O7—H7	0.818 (14)
Na2—Na1^v^	3.3978 (4)	O7—H8	0.857 (14)
S1—O1	1.4637 (5)	O8—Na2^viii^	2.3718 (6)
S1—O2	1.4700 (5)	O8—H9	0.813 (15)
S1—O3	1.4818 (5)	O8—H10	0.775 (15)
			
O1^i^—Na1—O4	93.81 (2)	O1^i^—Na2—Na1^v^	87.607 (15)
O1^i^—Na1—O5^ii^	167.57 (2)	O7^v^—Na2—Na1^v^	45.635 (14)
O4—Na1—O5^ii^	85.708 (19)	S2^vi^—Na2—Na1^v^	85.025 (8)
O1^i^—Na1—O6	83.704 (19)	S1^i^—Na2—Na1^v^	67.409 (7)
O4—Na1—O6	92.58 (2)	O6—Na2—Na1	43.196 (14)
O5^ii^—Na1—O6	83.912 (18)	O5—Na2—Na1	95.577 (15)
O1^i^—Na1—O2^iii^	105.32 (2)	O8^iv^—Na2—Na1	104.229 (16)
O4—Na1—O2^iii^	106.02 (2)	O1^i^—Na2—Na1	42.288 (13)
O5^ii^—Na1—O2^iii^	86.693 (19)	O7^v^—Na2—Na1	144.062 (17)
O6—Na1—O2^iii^	158.46 (2)	S2^vi^—Na2—Na1	137.059 (10)
O1^i^—Na1—O7	93.07 (2)	S1^i^—Na2—Na1	63.609 (7)
O4—Na1—O7	169.96 (2)	Na1^v^—Na2—Na1	118.260 (11)
O5^ii^—Na1—O7	85.995 (19)	O1—S1—O2	111.17 (3)
O6—Na1—O7	80.912 (19)	O1—S1—O3	111.30 (3)
O2^iii^—Na1—O7	79.125 (19)	O2—S1—O3	109.48 (3)
O1^i^—Na1—Na2^ii^	138.025 (17)	O1—S1—S2	108.60 (2)
O4—Na1—Na2^ii^	128.156 (17)	O2—S1—S2	109.04 (2)
O5^ii^—Na1—Na2^ii^	43.834 (13)	O3—S1—S2	107.13 (2)
O6—Na1—Na2^ii^	92.705 (15)	O1—S1—Na2^vi^	38.16 (2)
O2^iii^—Na1—Na2^ii^	67.367 (14)	O2—S1—Na2^vi^	75.51 (2)
O7—Na1—Na2^ii^	45.307 (14)	O3—S1—Na2^vi^	138.89 (2)
O1^i^—Na1—Na2	42.885 (14)	S2—S1—Na2^vi^	109.377 (8)
O4—Na1—Na2	98.391 (16)	S1—S2—Na2^i^	114.600 (8)
O5^ii^—Na1—Na2	124.870 (16)	S1—O1—Na1^vi^	141.35 (3)
O6—Na1—Na2	41.148 (13)	S1—O1—Na2^vi^	119.72 (3)
O2^iii^—Na1—Na2	141.592 (17)	Na1^vi^—O1—Na2^vi^	94.828 (18)
O7—Na1—Na2	81.785 (15)	S1—O2—Na1^vii^	149.10 (3)
Na2^ii^—Na1—Na2	118.261 (11)	Na1—O4—H1	120.6 (10)
O6—Na2—O5	87.88 (2)	Na1—O4—H2	111.8 (8)
O6—Na2—O8^iv^	97.82 (2)	H1—O4—H2	102.6 (12)
O5—Na2—O8^iv^	156.45 (2)	Na2—O5—Na1^v^	90.905 (19)
O6—Na2—O1^i^	85.146 (19)	Na2—O5—H3	124.4 (9)
O5—Na2—O1^i^	94.10 (2)	Na1^v^—O5—H3	110.7 (8)
O8^iv^—Na2—O1^i^	109.10 (2)	Na2—O5—H4	110.0 (9)
O6—Na2—O7^v^	171.97 (2)	Na1^v^—O5—H4	119.5 (9)
O5—Na2—O7^v^	87.654 (19)	H3—O5—H4	102.6 (12)
O8^iv^—Na2—O7^v^	83.76 (2)	Na2—O6—Na1	95.66 (2)
O1^i^—Na2—O7^v^	101.82 (2)	Na2—O6—H5	116.3 (9)
O6—Na2—S2^vi^	94.040 (15)	Na1—O6—H5	111.7 (9)
O5—Na2—S2^vi^	75.245 (14)	Na2—O6—H6	126.7 (9)
O8^iv^—Na2—S2^vi^	81.549 (16)	Na1—O6—H6	103.2 (9)
O1^i^—Na2—S2^vi^	169.337 (17)	H5—O6—H6	102.2 (12)
O7^v^—Na2—S2^vi^	78.368 (15)	Na2^ii^—O7—Na1	89.058 (19)
O6—Na2—S1^i^	105.273 (16)	Na2^ii^—O7—H7	107.5 (9)
O5—Na2—S1^i^	85.774 (14)	Na1—O7—H7	136.6 (9)
O8^iv^—Na2—S1^i^	114.306 (17)	Na2^ii^—O7—H8	126.5 (9)
O1^i^—Na2—S1^i^	22.118 (12)	Na1—O7—H8	97.7 (9)
O7^v^—Na2—S1^i^	81.039 (15)	H7—O7—H8	103.1 (12)
S2^vi^—Na2—S1^i^	152.391 (10)	Na2^viii^—O8—H9	110.1 (10)
O6—Na2—Na1^v^	131.833 (17)	Na2^viii^—O8—H10	130.1 (10)
O5—Na2—Na1^v^	45.260 (14)	H9—O8—H10	105.0 (13)
O8^iv^—Na2—Na1^v^	129.328 (17)		

Symmetry codes: (i) *x*, −*y*+1/2, *z*−1/2; (ii) *x*+1, *y*, *z*; (iii) *x*+1, −*y*+1/2, *z*−1/2; (iv) −*x*+1, *y*+1/2, −*z*+1/2; (v) *x*−1, *y*, *z*; (vi) *x*, −*y*+1/2, *z*+1/2; (vii) *x*−1, −*y*+1/2, *z*+1/2; (viii) −*x*+1, *y*−1/2, −*z*+1/2.

### (Na2S2O3H2O5_200K) Hydrogen-bond geometry (Å, º)

**Table:**

*D*—H···*A*	*D*—H	H···*A*	*D*···*A*	*D*—H···*A*
O4—H1···O3	0.774 (15)	2.045 (15)	2.8161 (7)	174.2 (14)
O4—H2···O2^ii^	0.820 (12)	2.022 (12)	2.8290 (7)	168.0 (11)
O5—H3···O3	0.836 (13)	1.961 (13)	2.7937 (7)	173.8 (12)
O5—H4···O3^vi^	0.786 (13)	2.109 (13)	2.8811 (7)	167.3 (12)
O6—H5···O4^vi^	0.859 (14)	1.984 (14)	2.8423 (8)	176.3 (13)
O6—H6···S2^ix^	0.860 (14)	2.495 (14)	3.3488 (5)	171.7 (12)
O7—H7···S2^iv^	0.818 (14)	2.531 (14)	3.3365 (5)	168.3 (12)
O7—H8···O8^i^	0.857 (14)	1.977 (14)	2.8334 (8)	177.3 (13)
O8—H9···S2^ii^	0.813 (15)	2.544 (15)	3.3245 (6)	161.3 (13)
O8—H10···S2	0.775 (15)	2.583 (15)	3.3499 (5)	171.0 (14)

Symmetry codes: (i) *x*, −*y*+1/2, *z*−1/2; (ii) *x*+1, *y*, *z*; (iv) −*x*+1, *y*+1/2, −*z*+1/2; (vi) *x*, −*y*+1/2, *z*+1/2; (ix) *x*+1, −*y*+1/2, *z*+1/2.
